# Novel *NEK8* Mutations Cause Severe Syndromic Renal Cystic Dysplasia through YAP Dysregulation

**DOI:** 10.1371/journal.pgen.1005894

**Published:** 2016-03-11

**Authors:** Valentina Grampa, Marion Delous, Mohamad Zaidan, Gweltas Odye, Sophie Thomas, Nadia Elkhartoufi, Emilie Filhol, Olivier Niel, Flora Silbermann, Corinne Lebreton, Sophie Collardeau-Frachon, Isabelle Rouvet, Jean-Luc Alessandri, Louise Devisme, Anne Dieux-Coeslier, Marie-Pierre Cordier, Yline Capri, Suonavy Khung-Savatovsky, Sabine Sigaudy, Rémi Salomon, Corinne Antignac, Marie-Claire Gubler, Alexandre Benmerah, Fabiola Terzi, Tania Attié-Bitach, Cécile Jeanpierre, Sophie Saunier

**Affiliations:** 1 INSERM UMR1163, Laboratory of Inherited Kidney Diseases, Necker-Enfants Malades Hospital, Paris, France; 2 Paris Descartes—Sorbonne Paris Cité University, Imagine Institute, Paris, France; 3 INSERM U1151, CNRS UMR8253, Paris Descartes—Sorbonne Paris Cité University, Necker-Enfants Malades Institute, Mechanisms and Therapeutic Strategies of Chronic Kidney Diseases, Necker Hospital, Paris, France; 4 INSERM UMR1163, Laboratory of Embryology and Genetics of Congenital Malformations, Paris, France; 5 Department of Genetics, AP-HP, Necker Hospital, Paris, France; 6 Department of Pediatric Nephrology, AP-HP, Robert Debré Hospital, Paris, France; 7 INSERM UMR1163, Laboratory of Intestinal Immunity, Paris, France; 8 Department of Pathology, Hospices Civils de Lyon, CHU de Lyon, Lyon, France; 9 Cellular Biotechnology Department and Biobank, Hospices Civils de Lyon, CHU de Lyon, Lyon, France; 10 CHU de La Réunion Saint-Denis/Saint-Pierre, La Réunion, France; 11 Anatomopathological Department, CHRU Lille, University Hospital, Lille, France; 12 Department of Clinical Genetics, CHRU Lille, Lille, France; 13 Department of Genetics, Femme Mère-Enfant Hospital, University of Lyon 1, Bron, France; 14 Department of Genetics, CHU Robert-Debré, Paris, France; 15 Fetal Pathology department, CHU Robert-Debré, Paris, France; 16 Multidisciplinary Department of Prenatal Diagnosis, La Timone Children’s Hospital, Marseille, France; 17 Department of Pediatric Nephrology, AP-HP, Necker Hospital, Paris, France; 18 Department of Histology-Embryology and Cytogenetics, AP-HP, Necker Hospital, Paris, France; Seattle Children's Research Institute, UNITED STATES

## Abstract

Ciliopathies are a group of genetic multi-systemic disorders related to dysfunction of the primary cilium, a sensory organelle present at the cell surface that regulates key signaling pathways during development and tissue homeostasis. In order to identify novel genes whose mutations would cause severe developmental ciliopathies, >500 patients/fetuses were analyzed by a targeted high throughput sequencing approach allowing exome sequencing of >1200 ciliary genes. *NEK8/NPHP9* mutations were identified in five cases with severe overlapping phenotypes including renal cystic dysplasia/hypodysplasia, *situs inversus*, cardiopathy with hypertrophic septum and bile duct paucity. These cases highlight a genotype-phenotype correlation, with missense and nonsense mutations associated with hypodysplasia and enlarged cystic organs, respectively. Functional analyses of *NEK8* mutations in patient fibroblasts and mIMCD3 cells showed that these mutations differentially affect ciliogenesis, proliferation/apoptosis/DNA damage response, as well as epithelial morphogenesis. Notably, missense mutations exacerbated some of the defects due to *NEK8* loss of function, highlighting their likely gain-of-function effect. We also showed that *NEK8* missense and loss-of-function mutations differentially affect the regulation of the main Hippo signaling effector, YAP, as well as the expression of its target genes in patient fibroblasts and renal cells. YAP imbalance was also observed in enlarged spheroids of *Nek8*-invalidated renal epithelial cells grown in 3D culture, as well as in cystic kidneys of *Jck* mice. Moreover, co-injection of *nek8* MO with WT or mutated *NEK8-GFP* RNA in zebrafish embryos led to shortened dorsally curved body axis, similar to embryos injected with human *YAP* RNA. Finally, treatment with Verteporfin, an inhibitor of YAP transcriptional activity, partially rescued the 3D spheroid defects of *Nek8*-invalidated cells and the abnormalities of NEK8-overexpressing zebrafish embryos. Altogether, our study demonstrates that *NEK8* human mutations cause major organ developmental defects due to altered ciliogenesis and cell differentiation/proliferation through deregulation of the Hippo pathway.

## Author Summary

Genes mutated in ciliopathies encode proteins with various localizations and functions at the primary cilium. Here we report novel *NEK8* mutations in patients with renal cystic hypodysplasia and associated ciliopathy defects. NEK8 belongs to a protein complex defining the Inversin compartment of the cilium. It is also a negative regulator of the Hippo signaling pathway that controls organ growth. We report genotype-phenotype correlation in the patients. We functionally demonstrate that the two types of mutations (missense *versus* nonsense) differentially affect ciliogenesis, cell apoptosis and epithelialisation. We also show that all the mutations lead to dysregulation of the Hippo pathway through nuclear YAP imbalance but that the nature of this imbalance is different according to the type of mutation. We confirm alteration of the Hippo pathway associated with *Nek8* mutation *in vivo* in *Jck* mice. Remarkably, we show that morphogenesis defects observed in *Nek8* knockdown epithelial cells or zebrafish embryos are rescued by Verteporfin, a specific inhibitor of YAP transcriptional activity, demonstrating the causative role of YAP dysregulation in the occurrence of these defects. Altogether, this study links *NEK8* mutations to dysregulation of the Hippo pathway and provide molecular clues to understand the variability of the multiorgan defects in the patients.

## Introduction

Ciliopathies are a group of autosomal recessive disorders caused by a dysfunction of the primary cilium. These conditions are multisystemic disorders, affecting left-right symmetry (*situs inversus*) and various organs such as retina (retinitis pigmentosa, Senior-Løken syndrome), brain (cerebellar vermis aplasia, Joubert syndrome), liver (cysts, intrahepatic biliary fibroadenomatosis), pancreas (cysts) as well as skeleton (cone shape epiphysis, narrow thorax, polydactyly), and/or kidney (renal cystic dysplasia (RCD), nephronophthisis (NPH)) [1, 2]. RCD and NPH are major genetic causes of end stage renal failure in children and perinatal death for RCD. RCD is a kidney developmental defect whose antenatal diagnosis by ultrasound examination reveals hyperechogenic kidneys. Phenotypes range from enlarged cystic dysplastic kidneys to undersized, hypodysplastic kidneys. RCD is usually classified among the spectrum of CAKUT (congenital anomalies of the kidney and urinary tract). NPH is characterized by atrophic kidney tubules with thickened basal membrane, interstitial fibrosis and, at a later stage, the development of cysts at the cortico-medullary junctions. Kidney size can be normal or reduced.

The primary cilium is a microtubule-based antenna-like structure present at the cell surface of almost all vertebrate cells, which controls signaling pathways (Hedgehog, canonical Wnt and planar cell polarity (Wnt/PCP)) with a major role during development and homeostasis of the kidney and other organs. In renal tubular cells, the primary cilium functions as a mechano/chemo-sensor regulating cell cycle and PCP in response to urine flow, in order to control the orientation of the mitotic spindles along the axis of the elongating tubules and the organization of the epithelial cells with respect to their neighbors in the tissue. Defects in these processes result in cyst formation [3]. Ciliopathies are genetically heterogeneous diseases and mutations in >100 genes encoding ciliary proteins have been identified in affected patients [4]. Genotype-phenotype correlation analyses revealed that different mutations in the same gene could result in phenotypes with varying severity. Among these genes, missense mutations in *NEK8/NPHP9* have been reported to lead to early onset isolated NPH [5]. However, a homozygous nonsense *NEK8* mutation leading to absence of the protein was also identified in a family with three fetuses presenting with a more severe phenotype similar to Ivemark I and II syndromes, characterized by enlarged cystic dysplastic kidneys, pancreas and liver, associated with skeletal abnormalities, asplenia and congenital heart defects [6]. NEK8 is a serine/threonine kinase composed of an N-terminal kinase domain and five C-terminal Regulator of Chromosome Condensation 1 (RCC1) repeat domains that belongs to the family of Never in Mitosis gene A (NIMA) proteins involved in the control of cell cycle progression [7]. In the cilium, NEK8 is located at the “Inversin (INVS) compartment”, a specific subcompartment of the proximal part of the axoneme, distal to the transition zone [8]. The function of this compartment is poorly understood, but human or mouse mutations in genes encoding components of the INVS compartment, *INVS/NPHP2*, *NPHP3* and *ANKS6/NPHP16*, are known to lead to infantile nephronophthisis with cystic kidneys, congenital heart defects and laterality defects [9–13]. Additionally, NEK8 is also present in the nucleus where it controls the replication fork progression during S-phase and regulates DNA damage response [14].

Recently, NEK8 has been proposed as a regulator of the Hippo signaling pathway [15]. The Hippo pathway regulates organ size by controlling the balance between cell proliferation and cell cycle arrest through the phosphorylation state and nuclear shuttling of transcriptional co-factors YAP/TAZ [16, 17]. Phosphorylation and nuclear shuttling of YAP/TAZ are strictly correlated to cell density, cell polarity and cellular actin cytoskeleton organization [18]. In low cell density conditions, YAP/TAZ are mainly unphosphorylated and able to translocate into the nucleus, resulting in cell proliferation. Conversely, at high cell density, YAP/TAZ are mainly phosphorylated and retained in the cytoplasm, leading to proliferation arrest. NEK8 has been reported to favor TAZ nuclear translocation, a process that is enhanced by NPHP4, encoded by another gene causing NPH, highlighting these two proteins as inhibitors of the Hippo pathway [15, 19].

Here, we report novel *NEK8/NPHP9* mutations in five unrelated cases with severe multisystemic phenotypes. This study highlights the dual phenotype associated with the nature of the mutations and the key functions of NEK8 in ciliogenesis and cell proliferation/differentiation through regulation of YAP.

## Results

### Novel *NEK8/NPHP9* mutations are associated with severe syndromic renal cystic dysplasia

**Fig 1:**
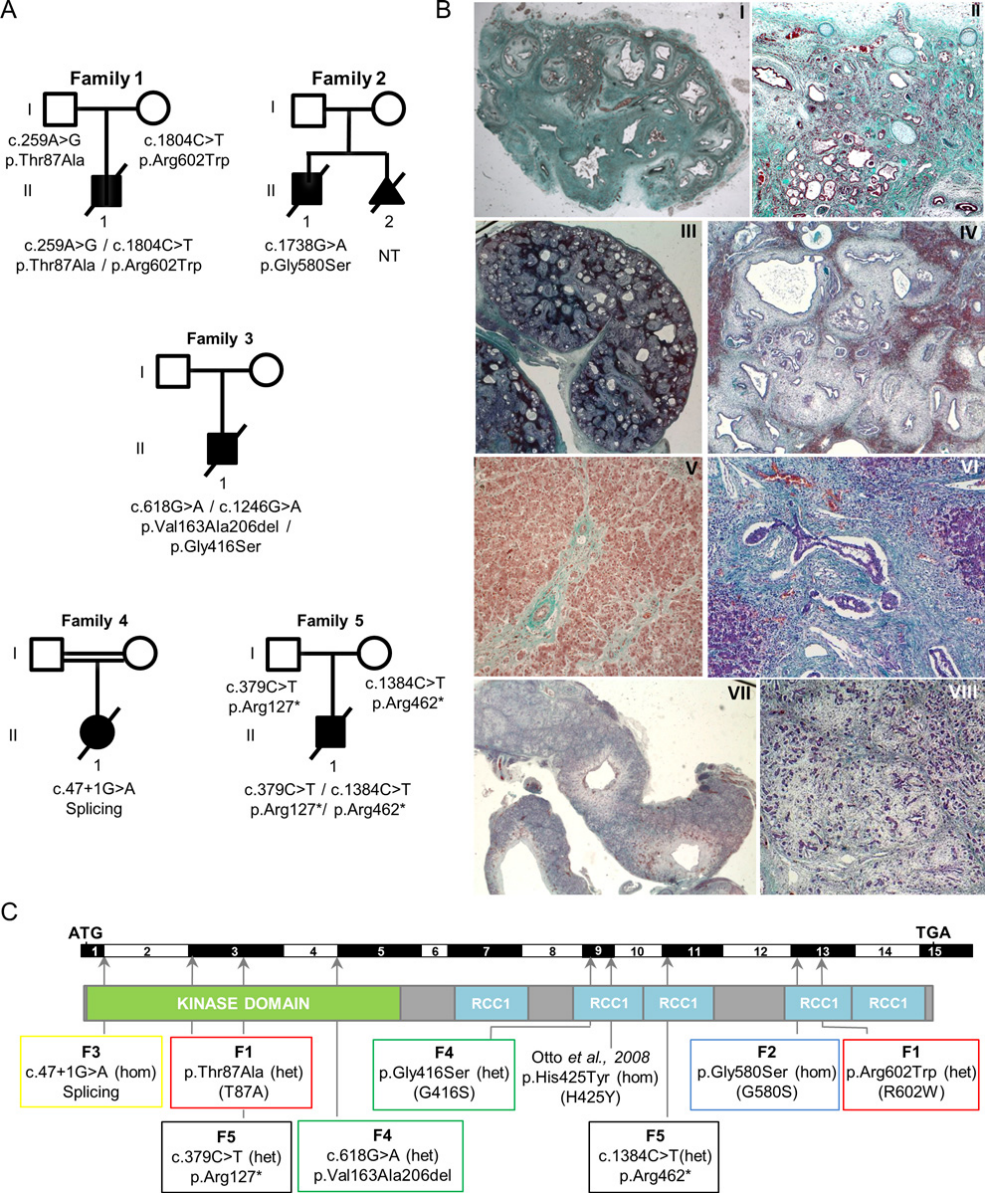
Identification of *NEK8/NPHP9* mutations associated with severe renal cystic dysplasia. (A) Pedigrees of the five families with identified compound heterozygous or homozygous *NEK8* mutations. (B) Trichrome light green staining of kidney (I-IV), liver (V, VI) and pancreas (VII, VIII) tissue sections from individual F1 II.1 (I, II, V) and F5 II.1 (III, IV, VI-VIII). (I-II) F1 II.1 presents a major cystic hypodysplasia of the right kidney (I) and a severe dysplasia of the left kidney (II) with persistence of few differentiated nephrons between large areas of fibrosis with primitive tubules (arrowhead) and presence of metaplastic cartilage (arrows). (III-IV) Fetus F5 II.1 shows enlarged cystic kidneys (III) of apparent normal shape. High magnification (IV) revealed a diffuse architectural disorganization with the presence of cysts of various sizes, fibrosis and dysplasic tubules (asterisks) and persistence of few glomeruli (arrowhead). (V-VIII) Portal tract fibrosis with extensions bridging adjacent portal areas (arrow) and hepatocytes focally dissociated by fibrosis (V) in affected individual F1 II.1. Fetus F5 II.1 presents with massive portal fibrosis with ductular proliferation (arrows) (VI) and large cystic structures in the pancreas (VII). Higher magnification reveals a massive and diffuse fibrosis with dissociation of the pancreatic parenchyma (VIII). Magnifications are 5x (III, VII), 10x (I), 40x (II, V, VIII), 100x (IV) and 200x (VI). (C) Location of the mutations: exons, position of the start (ATG) and stop (TGA) codons, as well as the position of the kinase and five RCC1 domains are indicated. Abbreviations: heterozygous (het); homozygous (hom). The mutation reported in a patient with nephronophthisis [5] is indicated. Mutations analyzed in functional studies are indicated with one letter nomenclature.

To identify novel mutations responsible for renal ciliopathies, we performed exon-enriched NGS targeting 1,222 genes associated with cilia structure/function, including all genes already known to be associated with ciliopathies (“ciliome sequencing”) [20–22] in two distinct cohorts of affected individuals: 342 patients with isolated or syndromic NPH and 200 fetuses or neonatal death cases with syndromic cystic dysplasia, including Meckel and Ivemark syndromes. Eight novel recessive mutations were identified in *NEK8/NPHP9* in five unrelated families with severe overlapping phenotypes (Fig 1A, Table 1).

**Table 1:** Clinical features of individuals with *NEK8* mutations.

Family	Renal phenotype	Extrarenal phenotype					Gender	Age at death	CS	Nucelotide alteration (segregation)	Protein Change	Exon or intron	PolyPhen2 / Sift score	Allele frequency in ExAc
		SI	Heart	Liver	Pancreas	Other								
F1	R: major cystic hypodysplasia ;	yes	Cardiomegaly, patent arteriosus ductus and interventricular septum hyperplasia	Hepatomegaly, cholestasis, paucity of bile ducts and fibrosis	/	/	M	1 month and 20 days	no	c.259 A>G (Het, P) c.1804 C>T (Het, M)	p.Thr87Ala p.Arg602Trp	Exon 3	0.882/0.00 0.991/0.00	Absent
	L: tubulointerstitial nephropathy with, dysplasia (primitive cystic tubules and cartilage nodules), cortical interstitial fibrosis											Exon 13		6.9.10^−5^
F2	R: microcysts in cortex and medulla;	No (yes in fetus sib)	Hypertrophiccardiomyopathy, interventricular septum hyperplasia	Paucity of bile ducts	/	Facial dysmorphy, narrow thorax, short bowed femurs, agenesis of corpus callosum	M	3 days	no	c.1783 G>A (Hom)	pGly580Ser	Exon 13	1 /0.00	8.2.10^−6^
	L: major hypodysplasia with macrocysts and cartilage nodules													
F3	R: hypodysplasia, incomplete corticomedullary differentiation, cartilage nodules and fibrosis;	yes	Cardiomegaly with intraventricular communication	Paucity of bile ducts	Short and fibrotic	Asplenia, narrow thorax, short bowed femurs	M	fetus*	no	c.618G>A (Het, M)	Splicing (p.Val163-Ala206del)	Exon 4	/	9.1.10^−5^
	L : absent									c.1246G>A (Het, P)	p.Gly416Ser	Exon 9	0.994/0.00	Absent
F4.	R / L: enlarged with cystic dysplasia and massive fibrosis	yes	/	/	Enlarged with cysts	Absence of uterus and vagina, gonads with abnormal morphology, agenesis of posterior vermis	F	fetus**	yes	c.47+1 G>A (Hom)	Splicing	Intron 1	/	Absent
F5	R / L: enlarged with cystic dysplasia incomplete, corticomedullary differentiation, primitive cystic tubules and luminal calcification, fibrosis	yes	/	Ductal plate anomalies with cystic biliary fibroadenomatosis	Enlarged with cysts	Facial dysmorphism, arthrogryposis (anamnios), neurological examination ND	M	fetus**	no	c.379C>T (Het, P)	p.Arg127*	Exon 3	/	Absent
										c.1384C>T (Het, M)	p.Arg462*	Exon 10	/	Absent

*NEK8/NPHP9* mutations are numbered according to the human cDNA (NM_178170.2). Position +1 corresponds to the A of ATG. Abbreviations are as follows: SI, *situs inversus*; CS, consanguinity; R, right kidney; L, left kidney; sib, sibling; P, paternal inheritance; M, maternal inheritance; Het, heterozygous, Hom, homozygous; ND, not determined. Termination of pregnancy at *26weeks, **28 weeks of gestation.

All five cases presented with kidney involvement associated with extra-renal defects including *situs inversus* (4 cases), cardiomegaly (3 cases), paucity of bile ducts (3 cases), pancreas defects (3 cases), narrow thorax and short bowed femurs (2 cases), and brain defects such as corpus callosum or vermis agenesis (2 cases) (Table 1). Patients/fetus from families 1, 2 and 3 shared developmental abnormalities including asymmetric renal hypodysplasia with one absent or extremely reduced in size kidney and the other one with major dysplasia, diffuse interstitial fibrosis and incomplete cortico-medullary differentiation, cardiac septal hyperplasia and liver alterations including paucity of bile ducts (Fig 1B and Table 1). The fetus from family 3, diagnosed with Ivemark I syndrome, presented with short pancreas and asplenia in addition to the defects listed above. The patient from family 2 and his affected sibling have already been reported [23]. In contrast, the fetuses from families 4 and 5 exhibited enlarged multicystic kidneys, cystic pancreas and liver, and agenesis of the vermis (fetus 4). The patient from family 1 carried compound heterozygous missense mutations: a paternally-inherited c.259A>G variation in exon 3 leading to a missense alteration in the kinase domain (p.Thr87Ala) and a maternally-inherited c.1804C>T variation in exon 13 resulting in an amino acid change in the RCC1 domain (p.Arg602Trp) (Figs 1A, 1C and S1A, Table 1). The patient from family 2 carried a homozygous missense mutation in exon 13 (c.1738 G>A) leading to an amino acid substitution (p.Gly580Ser) in the RCC1 domain (Figs 1A, 1C and S1A, Table 1). The fetus from family 3 carried a maternally-inherited heterozygous variant of the last base pair of exon 4 (c.618G>A) resulting in the in-frame skipping of exon 4 and loss of 44 amino acids in the kinase domain (p.Val163-Ala206del), associated with a paternally-inherited heterozygous variation (c.1246G>A) leading to a missense mutation (p.Gly416Ser) in the RCC1 domain (Figs 1A, 1C and S1A, Table 1). All four missense mutations were predicted as damaging by PolyPhen-2 and SIFT. The fetus from consanguineous family 4 carried a homozygous mutation affecting the first base of the 5' essential splice site in intron 1 (c.47+1 G>A). This mutation, which is predicted to totally abolish intron 1 splicing, is thus expected to lead to an absence of the protein. Finally, the fetus from family 5 carried two compound heterozygous nonsense mutations (paternally-inherited p.Arg127* and maternally-inherited p.Arg462*) that may result in truncated proteins and/or RNA decay as indicated by *NEK8* mRNA quantification (S1B Fig).

### NEK8 mutated proteins fail to localize to the ciliary axoneme in human primary fibroblasts and mIMCD3 cells and alter the INVS compartment composition

We first examined the impact of patient mutations on the targeting of NEK8 to the cilium as well as on ciliogenesis, using patient fibroblasts and mIMCD3 kidney cells.

**Fig 2:**
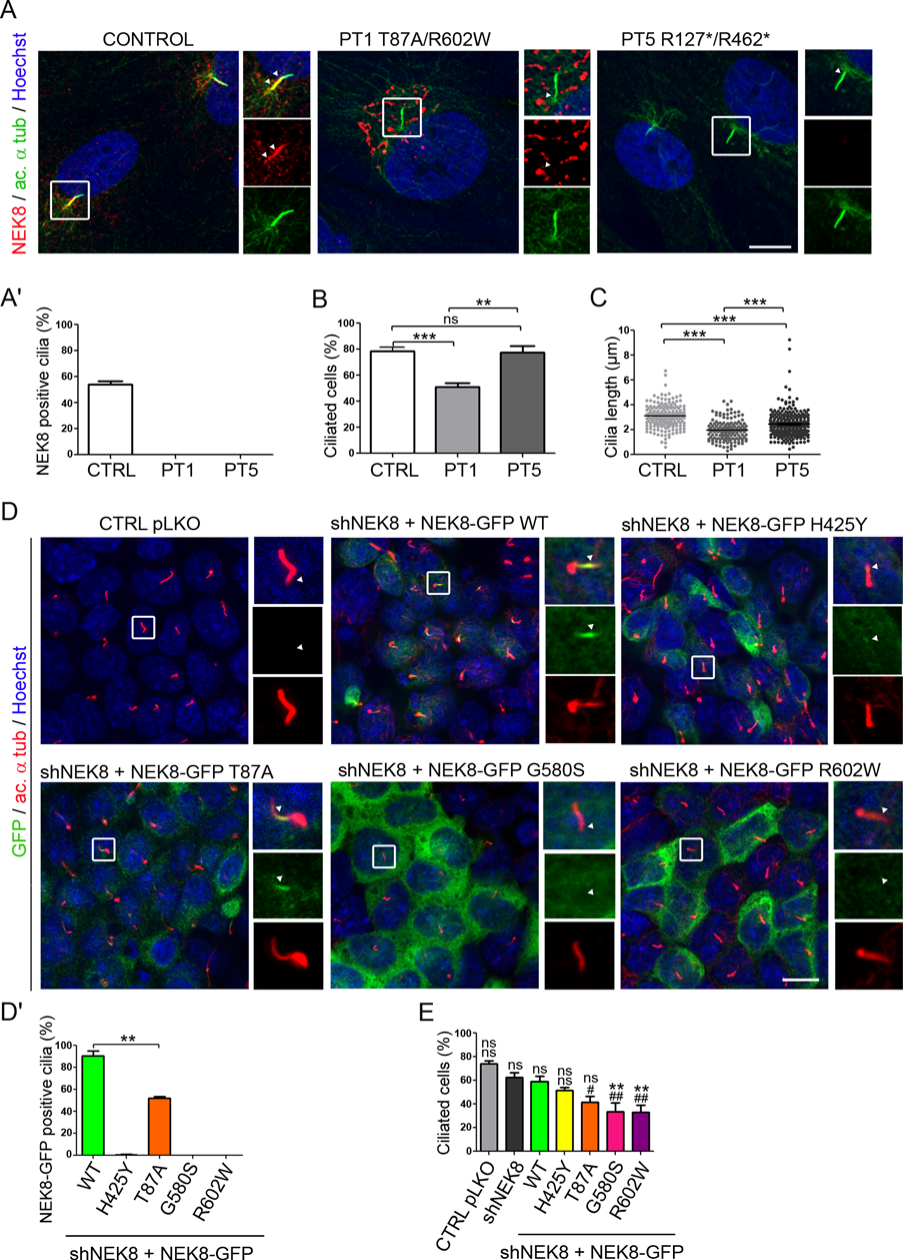
Mutant NEK8 proteins fail to localize at the ciliary axoneme in patient fibroblasts and in shNEK8 mIMCD3 cells re-expressing mutant constructs. (A) Fibroblasts from control (CTRL) and affected individuals II.1 of families 1 and 5 (PT1 and PT5) were grown for 6 days in standard medium followed by 2 days of serum starvation in order to induce cilia growth (high cell density). Cells were stained for endogenous NEK8 (red) and acetylated α-tubulin (green). Arrows (insets) indicate the presence and absence of NEK8 at the axoneme in control and patient fibroblasts respectively. Scale bar, 10 μm. (A’) Percentage of cells with NEK8 staining along the cilium in control and patient fibroblasts. (B-C) Graphs representing the percentage of ciliated cells (B) and cilia length (μm) (C) in control and patient fibroblasts. All graphs show the mean ± SEM of at least three independent experiments. ***p < 0.001, calculated via Kruskall-Wallis (B) or Bonferroni (C) post-hoc tests following ANOVA. (D) Ciliary localization of NEK8 in control pLKO and stable shNEK8 cells re-expressing GFP-tagged NEK8 wild type (WT) or patients’ variants. Cells were stained for acetylated α-tubulin (red, cilia), GFP (green) and nuclei (Hoechst, blue). Only NEK8-GFP WT and partially NEK8-GFP T87A are able to localize along the axoneme. Scale bar, 10 μm. (D’) Histogram showing the percentage of cells with positive NEK8-GFP staining at the primary cilium. (E) Graph showing the percentage of ciliated cells in shNEK8 and shNEK8 re-expressing the mutated proteins cells. Statistical analysis performed by Bonferroni post-hoc test following ANOVA, ns = non significant, *p < 0.05, **p < 0.01 when samples are compared to shNEK8+NEK-GFP WT and ^#^p < 0.05, ^##^p < 0.01to shNEK8.

We obtained primary fibroblasts from skin biopsies of family1 and 5 affected cases F1 II.1 and F5 II.1 (subsequently referred as PT1 and PT5) harboring compound heterozygous mutations p.T87A/p.R602W or p.Arg127*/p.Arg462*, respectively. While NEK8 was located in the proximal part of the ciliary axoneme in control ciliated fibroblasts, it was absent from cilia in PT1 fibroblasts carrying missense mutations (Fig 2A and 2A’), and was instead detected onto a cytoplasmic juxtanuclear vesicular compartment that we identified as the Golgi apparatus (S2A Fig). As an aside, we noted that endogeneous NEK8 or transfected NEK8-GFP proteins were also present at the Golgi in control fibroblasts (S2A and S2B Fig). However, in these cells, NEK8 Golgi localization seemed to be transient as it was clearly observed at low confluence (S2C Fig) and decreased in ciliated cells, suggesting a dynamic localization of NEK8 during ciliogenesis. In PT5 fibroblasts carrying nonsense mutations, no specific NEK8 staining was detected, indicating that the truncating mutations result in the loss of protein expression. Subsequent evaluation of the impact of *NEK8* mutations on ciliogenesis showed that the percentage of ciliated cells was significantly decreased in PT1 fibroblasts compared to control cells (50% *vs* 80%; Fig 2B). In contrast, ciliogenesis was not affected in PT5 fibroblasts (Fig 2B), in agreement with previously reported data showing that renal and MEF cells from *Nek8* knockout mice do not show ciliogenesis defects (10,25). In addition, cilia length was more reduced in cells with missense mutations than in those with loss-of-function mutations (Fig 2C).

In order to characterize the specific effect of each *NEK8* missense mutation, we generated murine inner medullary collecting duct (mIMCD3) cells depleted for Nek8 expression using a lentiviral vector for shRNA expression. A 80% reduction of the mRNA level was obtained in the *Nek8* knock-down cell line (shNEK8) compared to the control cell line transduced with the empty vector (pLKO) leading to a non detectable expression of the protein by immunofluorescence and western blot analyses (S3A, S3B and S3D Fig). The shNEK8 cells were then stably transfected with human NEK8-GFP constructs encoding either the wild-type protein (WT) or the missense variants identified in the patients. In addition, the p.H425Y NEK8 variant, located in the RCC1 domain and previously associated with isolated infantile NPH, was used as a control as it has been reported to alter NEK8 localization at the cilium [5, 24]. The level of expression of human NEK8-GFP fusions was in the same range in all the cell lines (S3C and S3D Fig). The WT NEK8-GFP protein localized to the ciliary axoneme at the “inversin (INVS) compartment” (Fig 2D and 2D'). The proteins with mutations in the RCC1 domains, p.G580S and p.R602W, were no longer present at the cilium and accumulated into the cytosol, similar to the p.H425Y protein [5]. This is in agreement with the major role of the RCC1 domains in cilia localization [24]. In contrast, an axonemal staining, weaker than in control cells, was detected in half of the ciliated cells expressing the NEK8 protein mutated in the kinase domain (p.T87A) (Fig 2D and 2D'). This result is coherent with a previous study showing that some NEK8 mutations in the kinase domain and affecting the kinase activity do not prevent its localization to the cilia in cell culture and in mice [10]. However, the decreased localization of NEK8 T87A-GFP protein points to a role of the kinase domain, possibly independently of the kinase activity, in ciliary NEK8 targeting process.

Then, we evaluated ciliogenesis in the various mIMCD3 cell lines. As observed in PT5 fibroblasts, ciliogenesis was not significantly affected in shNEK8 cells (Fig 2E). However, expression of all but WT and p.H425Y NEK8-GFP proteins led to a reduced percentage of ciliated cells, similar to what we observed in PT1 fibroblasts (p.T87A/p.R602W), highlighting a “gain of function” effect of the missense mutations on this process (Fig 2E).

**Fig 3:**
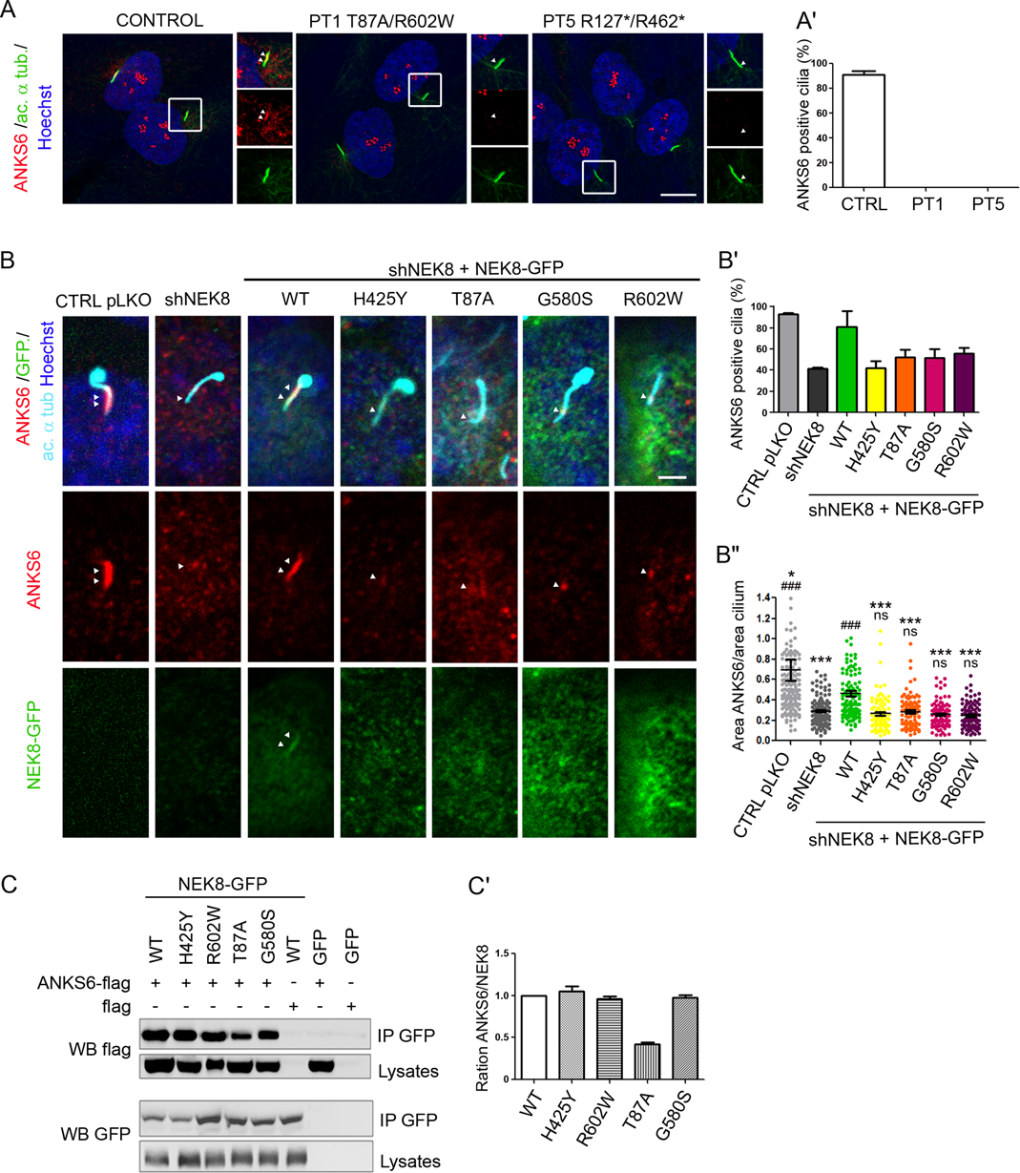
*NEK8* mutations prevent ANKS6 localization at the INVS compartment and interaction in patient fibroblasts and shNEK8 mIMCD3 cells. (A) Serum-starved fibroblasts from control (CTRL) and affected individual II.1 of families 1 and 5 (PT1 and PT5) were stained for ANKS6 (red) and acetylated α-tubulin (green). Insets are magnifications of ciliary axonemes, showing that ANKS6 staining is lost in patient fibroblasts. Scale bar, 10 μm. (A’) Graph (mean ± SEM of three independent experiments) representing the percentage of cells with ANKS6 staining at cilia in control and patient fibroblasts. (B) Control pLKO, shNEK8 and shNEK8 re-expressing WT or mutant NEK8-GFP cells were fixed after 5 days of culture and stained for ANKS6 (red), GFP (green), acetylated α-tubulin (cyan) and nuclei (Hoechst, blue). ANKS6 staining at the ciliary axoneme is decreased in shNEK8 mIMCD3 cells as well as in cells re-expressing the mutant constructs. Scale bar, 5 μm. (B’-B”) Graphs (mean ± SEM of three independent experiments) representing the percentage of cells with ANKS6 staining at cilia (B’) and the ratio between the areas of ANKS6 staining and total cilium (ANKS6/acetylated α-tubulin) (B”). Statistical analyses by Kruskal-Wallis post-hoc test following ANOVA, ns = non significant,*p < 0.05, ***p < 0.001 when samples are compared to shNEK8 + NEK8-GFP WT and ^###^p < 0.001 to shNEK8. (C) Lysates from HEK293T cells co-expressing GFP-tagged WT or mutant forms of NEK8 and Flag-ANKS6 were immunoprecipitated with an anti-GFP antibody. The co-immunoprecipitation of GFP-NEK8 and Flag-ANKS6 constructs was analysed by western-blot (WB) using GFP and Flag antibodies. (C’) Quantification of ANKS6 (Flag) *versus* NEK8 (GFP) band intensities showing that p.T87A NEK8 mutation decreases the interaction with ANKS6.

We next addressed the effect of NEK8 mutations on the ciliary localization of its partner ANKS6 [11]. While ANKS6 was located at the INVS compartment in 58% of control ciliated fibroblasts, its staining at the cilium was completely lost in PT1 and PT5 fibroblasts (Fig 3A and 3A’). As previously reported [10], a dramatic reduction of ANKS6 positive cilia was also observed in shNEK8 mIMCD3 cells (40% compared to 90% in control cells) (Fig 3B and 3B'). This phenotype was rescued by the re-expression of WT NEK8-GFP (80% of ANKS6-positive cilia), whereas none of the mutant forms were able to fully restore ANKS6 localization at the cilium (Fig 3B'). Moreover, in ANKS6 positive cilia of NEK8 mutant expressing cells, ANKS6 localization was restricted to the base of the cilium and did not extend along the INVS compartment as in control or NEK8-WT expressing cells (Fig 3B”). We next investigated the impact of NEK8 mutations on the interaction with ANKS6. Co-immunoprecipitation experiments demonstrated that only the p.T87A mutation led to a significant decrease of ANKS6 binding (Fig 3C and 3C’). This observation is in accordance with the recent report showing that NEK8 interacts directly with ANKS6 *via* its kinase domain [10, 11]. It is also in agreement with the observed lack of ciliary ANKS6 in shNEK8 cells expressing the p.T87A mutation. Altogether, these data demonstrate that all the NEK8 missense mutations affect both ciliogenesis and biogenesis of the INVS compartment in renal tubular cells and fibroblasts, by preventing correct targeting of NEK8 to cilia and/or binding to or recruitment of ANKS6.

### *NEK8* mutations affect cell cycle regulation

**Fig 4:**
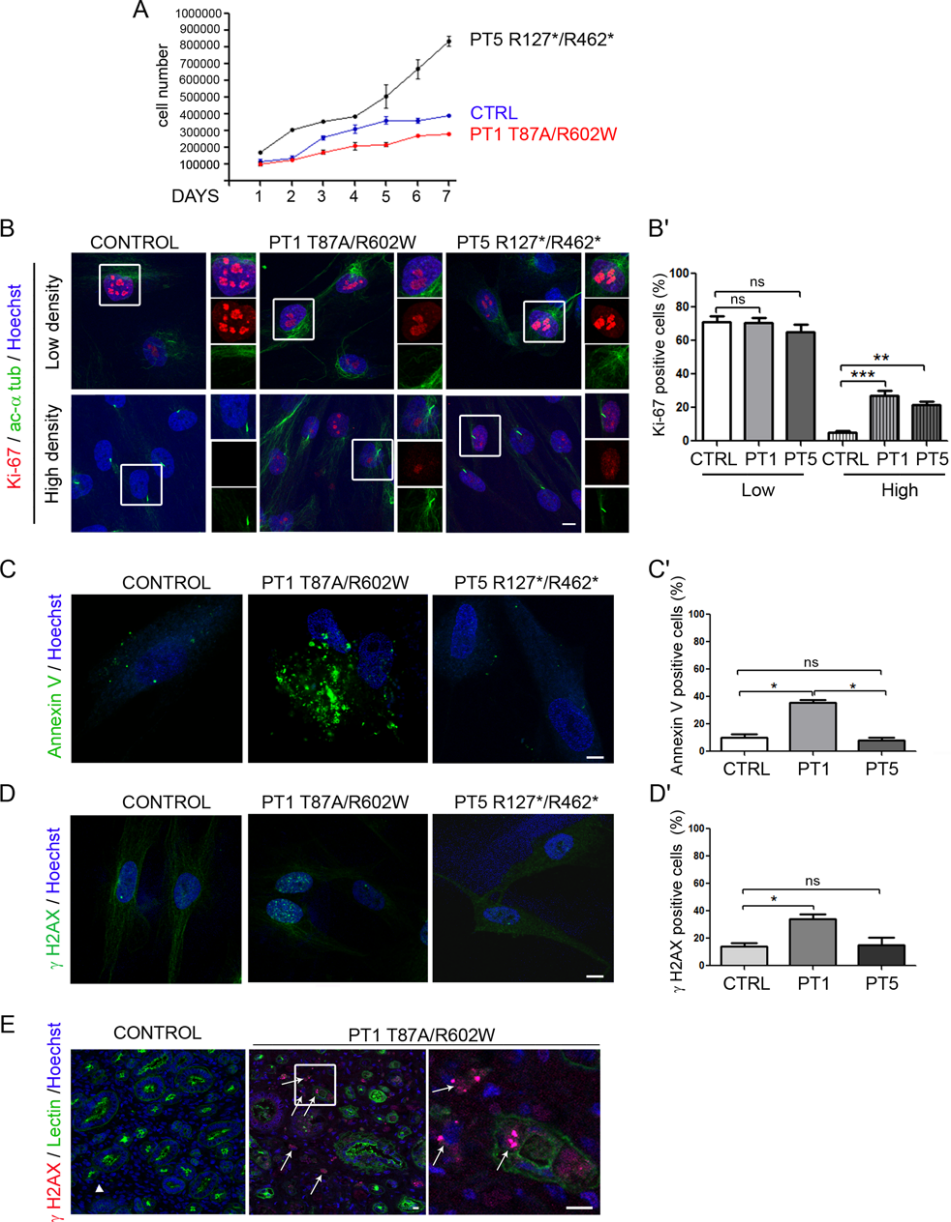
*NEK8* missense mutations cause increased apoptosis and DNA damage accumulation in patient fibroblasts. (A) Control (CTRL) and affected individual II.1 from families 1 and 5 (PT1 and PT5) fibroblasts cultured in standard medium were counted for 7 days. Cell population expansions of PT1 and PT5 fibroblasts were significantly slower and faster than control cells, respectively. (B) Immunostaining of Ki-67 (red, marker of proliferating cells) and acetylated α-tubulin (green, cilia) in proliferating (low density) and ciliated (high density) control and patient fibroblasts, showing Ki-67-positive ciliated patient cells. Scale bar, 10 μm. (B’) Percentage of Ki-67-positive cells in control and patient fibroblasts. ***p < 0.001, **p <0.01, calculated via Kruskal-Wallis test. (C) Immunolabelling of Annexin-V showing a higher rate of apoptosis in PT1 fibroblasts compared to control and PT5 cells. Scale bar, 10 μm. (C’) Graph representing the percentage of Annexin-V positive cells in control and patient fibroblasts. (D) Control and patient fibroblasts were grown for 2 days in normal medium (low cell density) and stained for γH2AX (red, marker of double strand breaks) and nuclei (Hoechst, blue). Scale bar, 10 μm. (D’) Quantification of the percentage of γH2AX-positive cells showing an increased staining only in PT1 cells. (E) PT1 kidney biopsy stained for γH2AX (red, marker of double strand breaks), peanut Lectin (green, tubules) and nuclei (Hoechst, blue). Arrows point out γH2AX-positive cells (inset) showing a dramatic increase of the staining. Scale bars, 10 μm. All graphs show the mean ± SEM of at least three independent experiments. *p < 0.05, calculated via Kruskal-Wallis test.

As NEK proteins are involved in cell cycle regulation [7], we sought to examine how *NEK8* mutations affect the cell cycle and proliferation. Counting the cell number over 7 days of culture revealed that patient fibroblasts behave differently depending on the type of mutation. While the population of PT1 cells with the missense mutations failed to expand as fast as control cells, PT5 fibroblasts with loss-of-function mutations expand much faster (Fig 4A). In order to characterize this difference, we analysed cell cycle progression of control and patient fibroblasts. At low cell confluence, Ki-67 staining, a marker of G1 to M phase, did not show any difference (Fig 4B). However, analysis of cell cycle by flow cytometry revealed a higher proportion of cells in S-phase in fibroblasts from patients compared to control, at the expense of cells in G0/G1 (S4 Fig). We also analysed Ki-67 staining at high cell confluence, when cells are ciliated. As expected, in this condition, most if not all control cells were Ki-67-negative, likely in quiescent G0 status. In contrast, ~20% of PT1 and PT5 cells remained Ki-67-positive, including ciliated cells (Fig 4B and 4B’; insets), indicating that patient cells fail to enter into G0. This was confirmed by flow cytometry analysis, showing an increased proportion of cells in G2/M for both patient cell lines (S4 Fig).

Altogether, these results indicate a dysregulation of the cell cycle in patient cells, likely resulting in increased cell proliferation. This is in agreement with the data obtained by counting PT5 cell number that increases over time; however, for PT1 fibroblasts, we observed by Annexin-V staining that they underwent more apoptosis than control and PT5 cells after 48 hours of culture (33% *vs* 15% of control and PT5 cells; Fig 4C and 4C'), explaining why the population PT1 do not expand as well as PT5 fibroblast cells.

NEK8, like other proteins involved in ciliopathies, has recently been described as a regulator of DNA damage response (DDR), with loss of NEK8 dramatically affecting S-phase progression upon DNA stress conditions [14]. Unlike the mentioned report, in non stress conditions we detected a significant increase in the proportion of nuclei positive for γH2AX (phosphorylated form of H2AX), a marker of DNA double-strand breaks [25], in PT1 fibroblasts compared to control and PT5 cells (40% *vs* 20% positive cells, respectively) (Fig 4D and 4D’). The increase of γH2AX staining was also detected in kidney sections from the same patient, compared to control kidney (Fig 4E). Hence, *NEK8* missense mutations cause defects in DNA repair, which may lead to apoptosis during cell proliferation.

### *NEK8* missense mutations cause abnormal 3D spheroid formation in mIMCD3 cells

**Fig 5:**
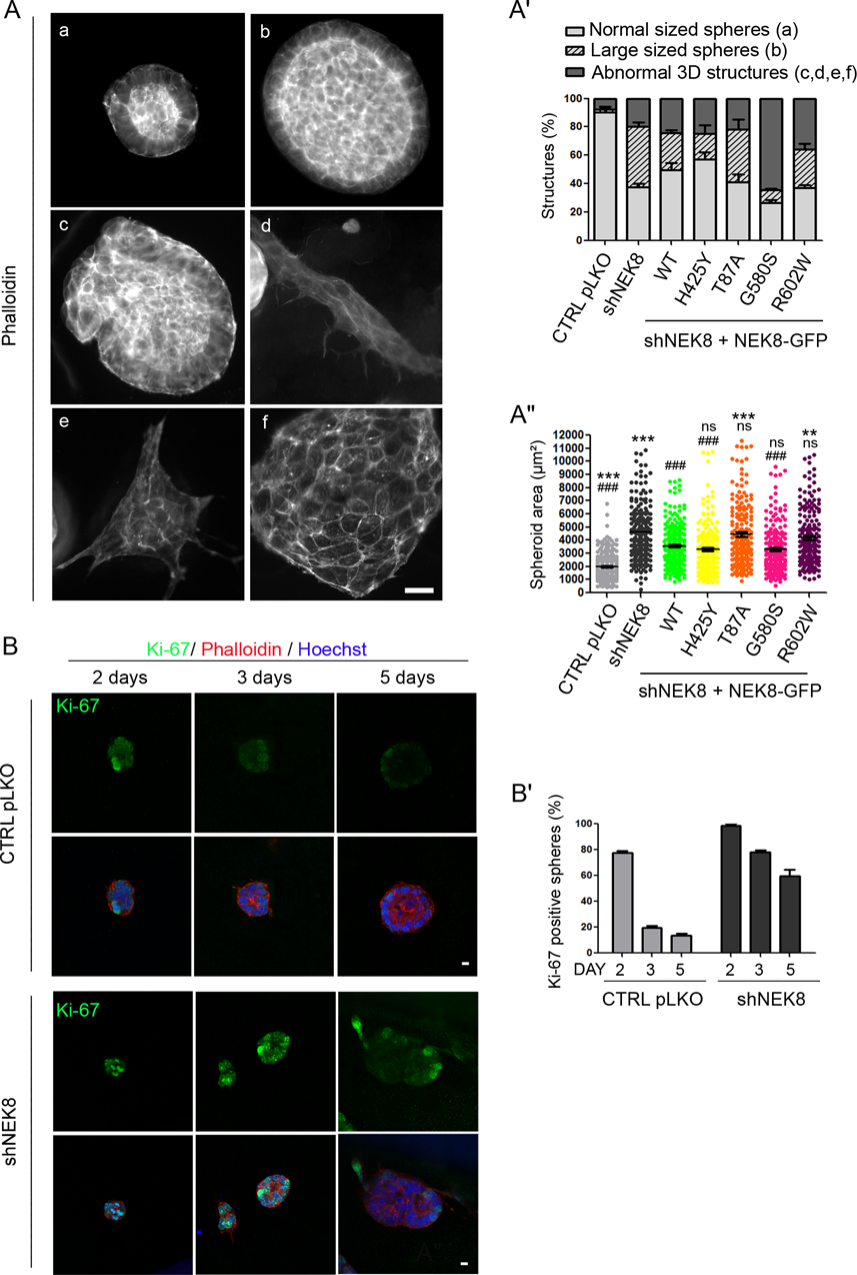
Abnormal 3D structures in shNEK8 and shNEK8 re-expressing NEK8 mutant protein cells. (A) 3D structures formed by shNEK8 and shNEK8 re-expressing NEK8 mutants. Cells were grown for 5 days in matrigel prior to phalloidin staining and six categories of structures were observed: (a) normal-sized spheres, (b) large-sized spheres, (c) malformed structures with lumen, (d) elongated structures, (e) branching structures and (f) aggregates without lumen. (A’-A”) Graphs (mean ± SEM of three independent experiments) showing the distribution of normal *versus* abnormal structures (A’) and quantification of the sphere areas (A”) for each cell line. Scale bar, 10 μm. Statistical analyses by Bonferroni post-hoc test following ANOVA, ns = non significant, **p < 0.01, ***p < 0.001 when samples are compared to shNEK8 + NEK8-GFP WT and ^###^p < 0.001 to shNEK8. (B). Three-dimensional (3D) spheroids derived from control pLKO and shNEK8 cells were fixed after 2, 3 and 5 days of culture, and were stained for Ki-67 (green), phalloidin (red, F-actin), Hoechst (blue, nuclei). Equatorial sections of representative spheres are shown. Scale bars, 10 μm. (B”) Quantification of Ki-67-positive spheres at 2, 3, and 5 days of culture.

In order to study the impact of *NEK8* mutations on epithelial organization, we performed 3D matrigel culture on mutated NEK8 re-expressing mIMCD3 cells [26]. As previously reported [14], 20% of the structures formed by shNEK8 cells were abnormal, without a clearly formed lumen (multi-cellular aggregates, tubular structures with excessive branching, malformed spheroids) after 5 days of culture (Fig 5A and 5A’). However, the most striking and previously unreported phenotype consisted of shNEK8 enlarged single lumen spheroids (Fig 5A, 5A’ and 5A”). These enlarged structures correlated with a sustained Ki-67 staining (Fig 5B and 5B’) during spheroid formation, as observed in patient fibroblasts in high cell density. Indeed, while at 2 days of culture the majority of control and shNEK8 spheroids were Ki-67-positive, at 5 days of culture only 10% of the structures remained Ki-67-positive in the control compared to 58% in shNEK8 spheroids. Expression of NEK8-WT protein, as well as p.H425Y form, partially rescued the proportion of normally-sized spheroids (Fig 5A’ and 5A”). In contrast, expression of the three other missense mutated proteins led to an increased proportion of malformed or tubular-shaped structures (p.G580S, p.R602W) (Fig 5A’) or failed to restore the correct size of the spheroids (p.T87A) (Fig 5A”). These results indicate that *in vitro* the loss of function of NEK8 leads to an overgrowth of the spheroids (enlarged spheroid structures), whereas the RCC1 missense mutations identified in the severe cases (p.G580S and p.R602W) affect epithelial morphogenesis, which may reflect the pathogenic effect of the different types of *NEK8* mutations during kidney development.

### *NEK8* mutations cause nuclear YAP imbalance in patient fibroblasts

**Fig 6:**
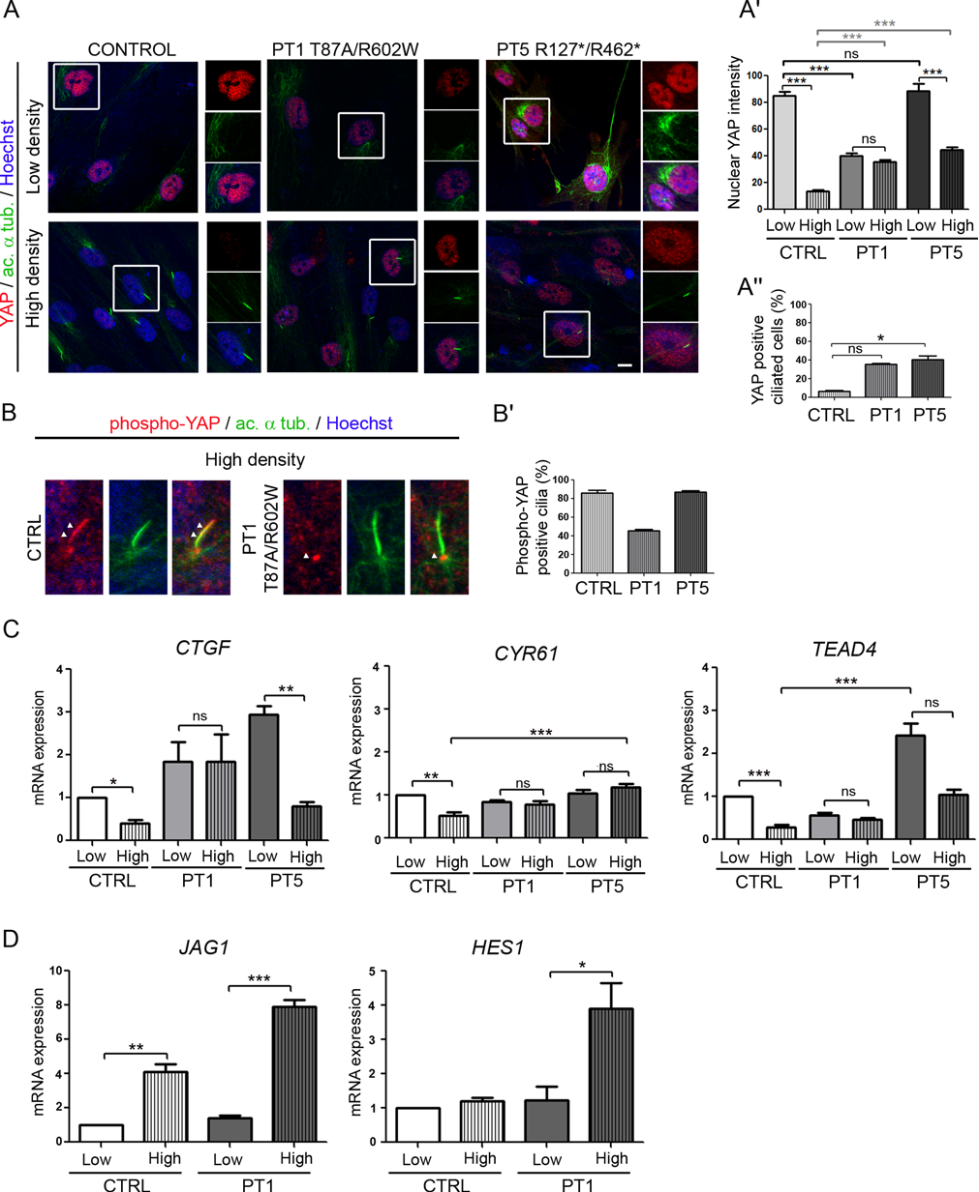
YAP nuclear shuttling is impaired in patient fibroblasts and results in Hippo pathway dysregulation. (A) Fibroblasts from control (CTRL) and affected individual II.1 of family 1 and 5 (PT1 and PT5) were grown for 2 days in standard medium with serum (low cell density) or for 6 days then serum-starved for 2 days (high cell density). Cells were co-immunostained for YAP (red), acetylated α-tubulin (green) and nuclei (Hoechst, blue). Insets show higher magnification of YAP localization. Scale bar, 10 μm. (A’, A”) Graphs (mean ± SEM of three independent experiments) showing the nuclear YAP intensity in control and patient fibroblasts (A’) and the percentage of ciliated cells positive for YAP staining (A”). ns = not significant, *p < 0.05, ***p <0.001, calculated by Bonferroni or Kruskal-Wallis post-hoc test following ANOVA, respectively. (B, B’) Localization of phospho-YAP at the cilium in control and patient fibroblasts (B) and quantification (mean ± SEM of three independent experiments) of phospho-YAP-positive cilia (B’). *p < 0.05 calculated via Kruskal-Wallis test. (C, D) qPCR analyses (mean ± SEM of three independent experiments) of the expression of YAP (*TEAD4*, *CYR61*, *CTGF*, *JAG1*) and Notch (*HES1*) target genes, normalized to *GAPDH*, in control and patient fibroblasts at low and high cell density. Statistical analyses were performed using Kruskal- Wallis test, ns = non significant, *p < 0.05, **p < 0.01, ***p < 0.001.

Since NEK8 has been reported as a regulator of the Hippo pathway, a critical pathway controlling growth and organ size [15], we investigated the impact of *NEK8* mutations on the regulation of this pathway. For this, we analyzed YAP localization and phosphorylation in low and high cell density, reflecting proliferating (YAP active) and quiescent (YAP inactive) states. As expected [18, 27], in control fibroblasts, YAP strongly accumulated into the nucleus in the low cell density condition (non ciliated cells), whereas it was hardly detectable in the nucleus when cells reached high cell density and became ciliated (Fig 6A, insets and Fig 6A’). In contrast, both patient cell lines present altered nuclear YAP staining. PT1 fibroblasts showed a weak nuclear YAP staining compared to control cells at low cell density and this expression level was not modulated by confluence (Fig 6A and 6A’). In contrast, a strong nuclear YAP staining was observed in PT5 fibroblasts at low cell density, that decreased at high cell density but remained higher than in control cells (Fig 6A and 6A'). Consequently, whereas the vast majority of control ciliated cells did not show any nuclear YAP staining (Fig 6A), maintenance of nuclear YAP staining occurred in about 40% of ciliated patient fibroblasts (Fig 6A, insets and Fig 6A”). These results indicate that *NEK8* mutations lead to an improper regulation of nuclear YAP shuttling from proliferation to quiescent state, which may affect cell growth and differentiation.

Upon cell confluence, inactivation of YAP is mediated by its phosphorylation on Ser127 [17]. As expected, immunofluorescence analysis revealed that cytosolic phospho-YAP increased with cell density in control fibroblasts (S5 Fig). A similar increase was observed in patient fibroblasts, indicating that *NEK8* mutations do not affect YAP phosphorylation (S5 Fig). YAP phosphorylation is regulated by the MTS1/2-SAV1 complex, which has also been recently reported to promote ciliogenesis and to localize at the basal body in ciliated cells [28]. We thus carefully examined phospho-YAP localization and detected the presence of phospho-YAP along the axoneme in 80% of cilia in control and PT5 fibroblasts (Fig 6B and 6B’). However, in PT1 fibroblasts, phospho-YAP was absent from cilia and the staining was restricted to the cilium base in half of the ciliated cells (Fig 6B and 6B’). Altogether, these data underline that NEK8 mutations differently impair the cell density regulated nucleocytoplasmic shuttling of YAP, whereas only the missense mutations alter the localization of phospho-YAP at the cilium.

In order to better understand how NEK8 mutations affect YAP nucleocytoplasmic shuttling, we analysed the ability of WT and mutated NEK8-GFP to promote nuclear YAP-myc localisation in co-transfected HEK393 cells. The co-transfection of each NEK8 mutant form with YAP-myc decreased the nuclear translocation of YAP-myc compared to WT NEK8-GFP, confirming the results observed in PT1 fibroblasts (S6A Fig). Moreover, proximity ligation assay performed on cells co-expressing YAP-myc and NEK8-GFP-WT revealed that the two proteins are in close vicinity and are likely to interact at the perinuclear region (S6B Fig), supporting a direct role of NEK8 in YAP regulation.

Then, in order to examine if nuclear YAP imbalance had an impact on target gene regulation, we analysed the expression of YAP target genes as well as the transcriptional YAP co-regulator TEAD4 in control and patient fibroblasts. In control cells, the expression of *CYR61*, *CTGF* and *TEAD4* was decreased in confluent cells *versus* non-confluent cells (Fig 6C), thus following the amount of nuclear YAP in these cells, as previously described [29]. In contrast, in PT1cells the expression of these genes was maintained at a similar level in high *versus* low confluent cells, consistent with maintenance of nuclear YAP localization in confluent cells. In PT5 cells, the expression of YAP target genes also reflected the nuclear YAP localization, with a high level of expression in non-confluent cells that decreased when cells reached confluence, although remaining at a higher level than in control cells.

Among the signalling pathways reported to be downstream of YAP, we examined the Notch pathway, crucial for kidney and liver development [30, 31]. In agreement with the upregulation of this pathway upon cell-cell contact, we observed an overexpression of *JAG1* as well as the downstream target *HES1* at high cell confluence in both control and PT1 fibroblasts. However, this increase was much higher in patient cells (Fig 6D), indicating that dysregulation of nuclear YAP may also affect Notch signaling.

### Yap and Hippo target genes are upregulated in *Jck* mice

**Fig 7:**
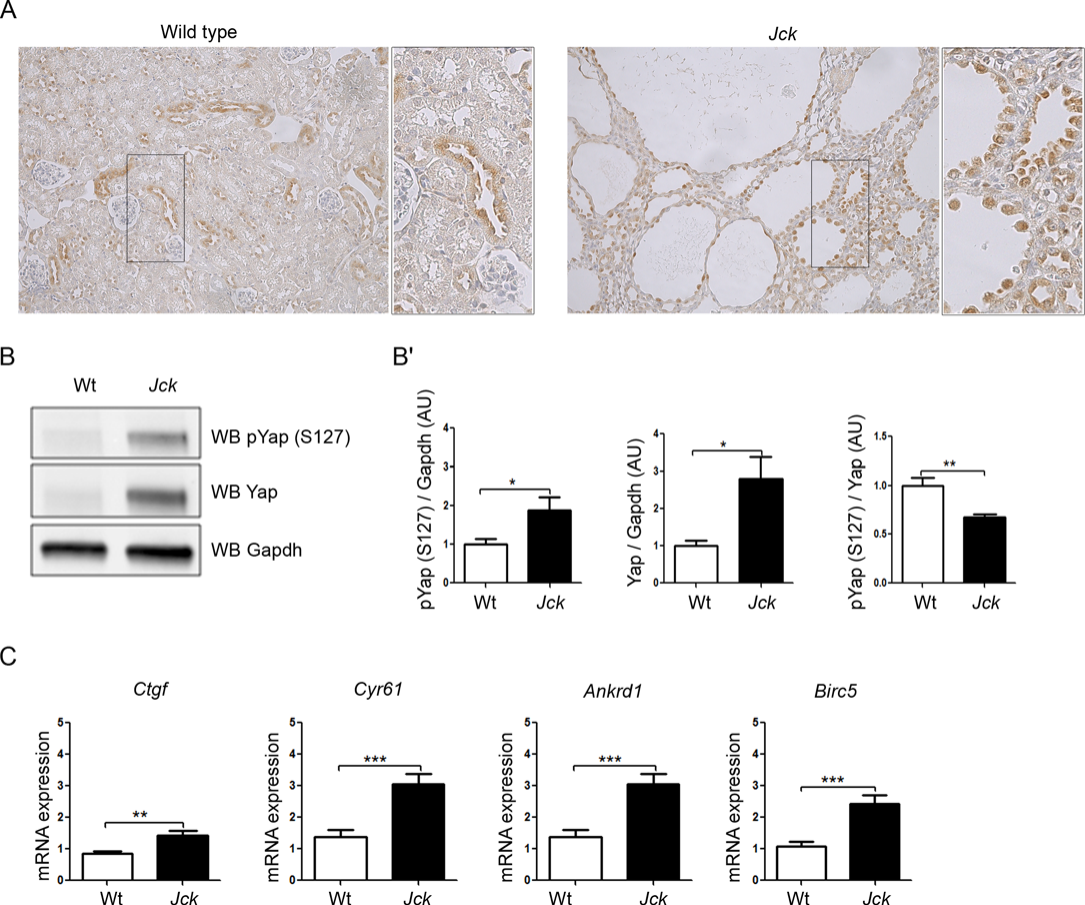
Yap and Yap target gene expression are induced in *Jck* mice. (A) Immunohistochemistry of Yap in wild-type (Wt) and *Jck* mice at 5 weeks of age (n = 4 of each). (B-B’) Western blot analysis (B) and quantification of YAP and phospho-YAP (S^127^) expression (B’) in Wt (n = 8) and *Jck* mice (n = 8) at 5 weeks of age. **p < 0.01, calculated by Mann-Whitney test. (C) Relative quantification of Yap targets *Ctgf*, *Cyr61*, *Ankrd1* and *Birc5* mRNA expression in wild-type (n = 10) and *Jck* mice (n = 8) at 5 weeks of age. **p < 0.01, ***p < 0.001, calculated by Mann-Whitney test.

In order to investigate if *NEK8* mutations also led to YAP activation *in vivo*, we studied Yap and target gene expression in mutant juvenile cystic kidney (*Jck*) mice which bear a missense p.G448V mutation in the highly conserved RCC1 domain of Nek8 [32, 33]. Immunohistochemistry assay showed that Yap expression was predominantly cytoplasmic in the kidneys of wild-type mice, whereas it was markedly increased in nuclei of kidneys of 5-week old *Jck* mice, an age at which these mice exhibited a polycystic kidney disease (Fig 7A). Yap staining was particularly intense in the nuclei of tubular epithelial cells lining the cysts (Fig 7A, insets). Western blot analysis of whole-kidney extracts (Fig 7B and 7B') confirmed that the expression of both Yap and phospho-Yap (S127) was increased in mutant mice. Nevertheless, the phospho-Yap (S127) / Yap ratio was decreased, pointing again to an upregulation of Yap activity. In line with these observations, quantitative RT-PCR confirmed that Yap target genes, i.e. *Ctgf*, *Cyr61*, *Ankrd1* and *Birc5* were upregulated in *Jck* mice (Fig 7C).

### Defects induced by *NEK8* alterations are rescued by Verteporfin treatment

**Fig 8:**
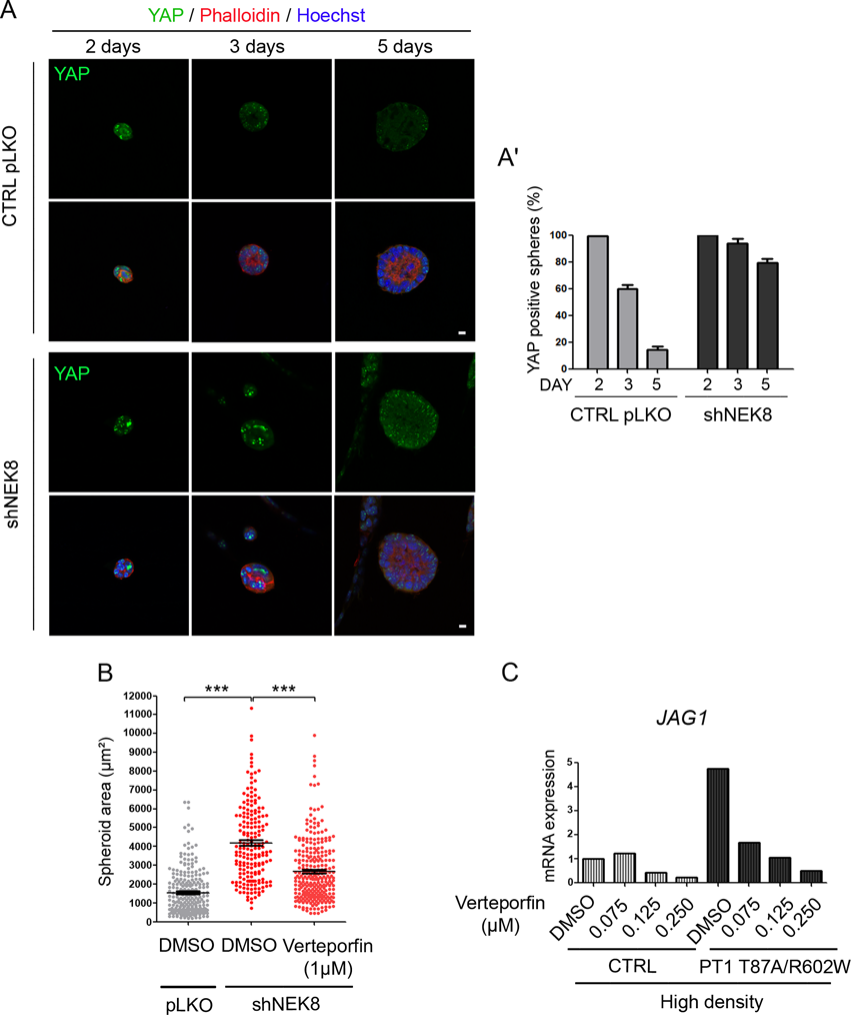
Nuclear YAP persistence in shNEK8 3D spheroids results in morphological and size abnormalities that are rescued by Verteporfin treatment. (A) Three-dimensional (3D) spheroids derived from control pLKO and shNEK8 cells were fixed after 2, 3 and 5 days of culture. Structures were stained for YAP (green), phalloidin (red, F-actin) and nuclei (Hoechst, blue). Equatorial sections of representative spheres are presented, showing that YAP is upregulated in shNEK8 3D spheres. Scale bars, 10 μm. (A’) Quantification of YAP-positive spheres at day 2, 3 and 5. (B) Graph showing the quantification of sphere areas after 5 days of culture, upon DMSO or Verteporfin treatment. Verteporfin treatment reduces the area of the shNEK8 spheroids. The graph indicates the mean **+/-** SEM. ***p < 0.001, calculated by Student *t*-*test* with Welsh correction. (C) qPCR analysis of *JAG1* expression in control (CTRL) and patient (PT1) fibroblasts, cultivated at high density and treated with different doses of Verteporfin as indicated. Data show a reduction in the expression of *JAG1* in a dose-dependent manner in patient fibroblasts.

To determine if the epithelial morphogenesis abnormalities observed in shNEK8 and mutated NEK8 re-expressing cells were caused by deregulation of the Hippo pathway, we quantified nuclear YAP staining during spheroid formation. Control and shNEK8 cells grown in 3D in matrigel were fixed after 2, 3 and 5 days of culture and stained for YAP (Fig 8A). After 2 days, the majority of control and shNEK8 spheroids were positive for YAP. In control cells, the proportion of YAP-positive spheroids dramatically decreased to 15% at day 5 (Fig 8A and 8A’). However, nuclear staining of YAP was still present in 75% of shNEK8 spheroids after 5 days of culture. The continuous activation of YAP in the nucleus in shNEK8 mIMCD3 cells could thus promote cell growth in forming structures, causing abnormal enlarged spheroids as recently described in MDCK cells [34]. To confirm this hypothesis, we performed size rescue experiments using Verteporfin, an inhibitor of YAP-TEAD4 interaction [35]. Indeed, we observed that 1 μM of Verteporfin caused a reduction in size of the spheroids formed by shNEK8 cells after 5 days (Fig 8B). In parallel, we confirmed that the increased expression of YAP targets observed in patient fibroblasts and shNEK8 IMCD3 cells was reduced with Verteporfin treatment (S7 Fig).

We also investigated if persistence of nuclear YAP in confluent patient fibroblasts was involved in the abnormal activation of the Notch pathway. As shown in Fig 8C, Verteporfin treatment performed in the high cell density condition dramatically reduced *JAG1* expression in patient fibroblasts, further demonstrating the link between YAP activation and Notch dysregulation in NEK8 mutant cells.

**Fig 9:**
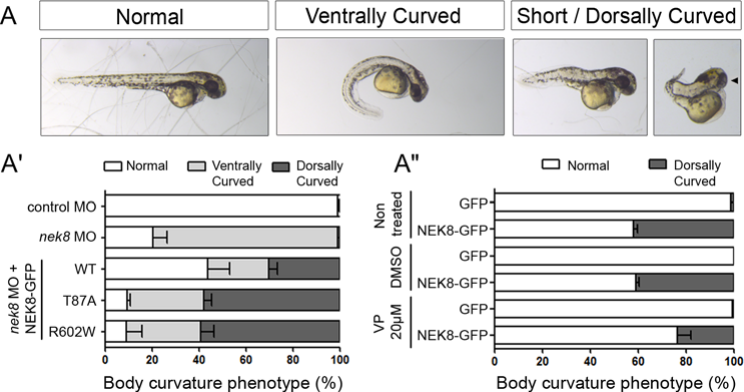
NEK8 overexpression in zebrafish induces Hippo pathway deregulation. (A) Representative pictures of the different classes of body phenotype observed upon *nek8* morpholino (MO) or human *NEK8-GFP* RNA injections in zebrafish embryos. Arrowhead points out cyclopia observed in a subset of short embryos. (A’) Graph showing the distribution of the three classes of body axis curvature observed in rescue experiments with WT or mutated (T87A, R602W) *NEK8-GFP* RNA forms. 30% of embryos co-injected with *nek8* MO and WT *NEK8-GFP* RNA exhibited a stunted dorsally curved body axis similar to embryos injected with human *YAP* RNA. Data are from three independent experiments (n = 90 to 180 embryos). (A”) Graph showing the distribution of normal and stunted dorsally curved animals among *GFP* or WT *NEK8-GFP* RNA injected embryos upon Verteporfin (VP) treatment. A partial rescue of the phenotype can be observed in the presence of 20 μM Verteporfin. Data are from four independent experiments (n = 120 to 240 embryos).

Finally, we examined the impact of *NEK8* mutations identified in patients in zebrafish, an *in vivo* model relevant for ciliopathies [36]. Embryos injected with *nek8* morpholino (MO) displayed the classical ciliopathy-related phenotype including curved body axis, laterality defects and pronephric cysts (Fig 9A and 9A’ and Fig 8A and 8A’), as previously described [37]. Body curvature was partially rescued by co-injection of human WT *NEK8-GFP* RNA (43% of normal embryos compared to 20% in *nek8* morphants; Fig 9A’) but not by mutated *NEK8-GFP* RNA (p.T87A and p.R602W), thus confirming the pathogenicity of the human missense mutations. Of note, we observed that co-injection of *nek8* MO with WT *NEK8-GFP* RNA led to shortened dorsally curved embryos with occasionally a unique centered eye (Fig 9A and 9A’), a phenotype that was exacerbated by NEK8 missense mutations (60% *vs* 30%), further indicating their gain of function effect. Overexpression of human NEK8 accounts for the shortened dorsally curved phenotype since it was observed in 40% of embryos injected with WT RNA only (Fig 9A”). We also observed laterality defects (70% of embryos) and pronephros abnormalities (cysts or developmental defects in 50% of embryos) upon human NEK8 overexpression (S8A and S8A’ Fig). As a similar dorsal curvature phenotype has been reported for embryos injected with human *YAP* RNA [38], we performed rescue experiments using Verteporfin treatment (Fig 9A”, S8 Fig). WT *NEK8-GFP* RNA-injected embryos were treated with 20 μM Verteporfin from 90% epiboly stage to 34 hours post fertilisation (hpf). Analysis of Yap target gene expression by qPCR revealed that human NEK8 overexpression does induce an upregulation of the target genes, which is blocked by Verteporfin treatment (S8B Fig). Contrary to laterality defects which remained unchanged, the proportion of stunted dorsally curved embryos and pronephros abnormalities decreased by 50% and 25% respectively upon treatment (Fig 9A”, S8A” Fig). These data indicate that the NEK8 overexpression-related phenotype partially results from an upregulation of YAP activity in zebrafish.

Altogether, these data demonstrate that abnormal YAP activation accounts for the epithelialisation, signaling and morphogenesis defects linked to *NEK8* mutations.

## Discussion

To date, only two recessive human *NEK8* mutations had been reported, one missense mutation in the RCC1 domain in a patient with early onset NPH and one nonsense mutation in the same domain in three fetuses from a consanguineous family with Ivemark I/II syndromes including cystic dysplastic lesions occurring in kidneys, liver and pancreas, and heart and skeletal defects [5, 6]. Here, we describe 8 novel *NEK8* mutations in five cases with severe multi-organ developmental defects, and the first association of *NEK8* mutations with renal hypodysplasia and agenesis, *situs inversus*, agenesis of the vermis and bile duct paucity. Based on our results, *NEK8* seems to be a major gene for renal dysplasia, since mutations were identified in 5 out of 200 analyzed families with dysplastic kidneys. Conversely, the previously identified mutation in a patient with infantile NPH seems to be a rare event, as we did not identify any other *NEK8* mutation among the 342 analyzed NPH families. We also report the first two human mutations in the serine/threonine kinase domain of the protein.

We observed a strong genotype-phenotype correlation. Fetuses with total NEK8 loss-of-function mutations (c.47+1G>A, family 3; p.R127*/p.R462*, family 5) presented enlarged cystic kidneys and pancreas associated with proliferative cystic biliary ducts, characteristics of Renal-Hepatic-Pancreatic Dysplasia syndrome (OMIM #208540), as described for the previously reported fetuses with a nonsense mutation [6]. In contrast, the three patients carrying missense mutations (p.T87A, p.R602W, p.G580S and p.G416S) or in-frame deletion due to a splicing defect (p.V163-A206del) presented with asymmetric dysplasic/hypodysplasic kidneys (agenesis in one case) with loss of differentiation, cortical interstitial fibrosis, dilated tubules and cartilage nodules, associated with paucity of bile ducts (Table 1).

The functional analyses of the *NEK8* mutations indicate that loss-of-function and missense mutations differentially alter ciliogenesis, proliferation/apoptosis and epithelial morphogenesis. Indeed missense mutations exacerbate some of the defects due to NEK8 loss of function both *in vitro* and *in vivo* (zebrafish), highlighting their likely gain of function effect. In particular, only missense mutations lead to prominent ciliogenesis defects with reduction of percentage of ciliated cells and cilia length in fibroblasts and mIMCD3 cells. While both types of mutations affect cell cycle regulation, missense mutations also alter the function of NEK8 as a regulator of DNA damage response [14], resulting in increased cell apoptosis in fibroblasts and kidney tissue. 3D culture assays showed that shNEK8 mIMCD3 cells (loss of function) form large spheroids (cystic phenotype) compared to NEK8 mutant form re-expressing mIMCD3 cells that mostly fail to get organized into spheroids (dysplastic phenotype). Finally, co-injection of human mutant *NEK8* RNA in *nek8* zebrafish morphants further enhances their severe morphological alterations, resulting in a shortened dorsally curved body axis. It is noteworthy that the missense mutation previously reported in a patient with infantile NPH (p.H425Y, [5]) does not have the same gain of function effect, thus explaining the less severe phenotype. Therefore, this genotype/phenotype correlation points out the dual function of NEK8 for which a loss of function (nonsense mutations) leads to proliferative/cystic phenotypes and a gain of function (missense mutations) to hypodysplastic phenotypes with loss of differentiation (S9 Fig).

Although *Nek8* mouse model phenotypes are different from those of human cases, a genotype-phenotype correlation seems to also exist in rodents in term of severity of the renal phenotypes. The *Nek8*^*jck/jck*^ mice, carrying a missense homozygous mutation in the RCC1 domain (p.G448V), which has been demonstrated to be a gain of function, develop enlarged cystic kidneys [32]. In contrast, *Nek8* knockout mice (*Nek8*^*tm1Bei*^) present a mild renal phenotype, with dilated proximal tubules and glomerular cysts [39] and the mouse model with a missense mutation (p.I124Y) in the kinase domain (*Nek8*^*roc*^) exhibits hydroureter, cystic tubular dilations and small glomerular cysts [10]. However, both *Nek8*^*tm1Bei*^ and *Nek8*^*roc*^ mice also present *situs inversus* and heart defects that lead to death at birth and which may prevent renal cyst formation during the final, post-birth steps of murine nephrogenesis. Association of kidney defects with *situs inversus* and heart defects was also observed in the five human cases. This phenotype is consistent with a general alteration of the ciliary function of NEK8 and the integrity of the INVS compartment (absence of NEK8 or ANKS6 proteins in the cilia). Indeed, mutations in genes encoding other components of the INVS compartment (*INVS/NPHP2*, *NPHP3* and *ANKS6/NPHP16*) are known to lead to infantile NPH associated with enlarged cystic kidneys or to kidney cystic dysplasia associated with congenital heart defects and *situs inversus* [9, 11–13]. Interestingly, we also identified a homozygous frameshift mutation (c.1010_1011del, p.G337Afs*16, family 6) in *ANKS6* (S10 Fig) in a fetus whose phenotype was similar to that of *NEK8* loss of function cases, i.e. enlarged cystic kidneys associated with enlarged fibrotic pancreas, *situs inversus* and cardiopathy. This is in agreement with the phenotype of *Anks6*^*Streaker*^ mice whose *Anks6* mutation (p.M187K) decreases the binding to and activation of Nek8 and leads to cystic kidneys, *situs inversus* and congenital heart defects, thus mimicking the phenotype of patients with *NEK8* mutations [10]. Identification of this *ANKS6* mutation, together with our functional data, strengthens the close relationship between NEK8 and ANKS6, i.e. NEK8 recruits its target ANKS6 to the cilium, which in return enhances NEK8 kinase activity [10].

Besides its function at cilia, NEK8 is critical for cell cycle regulation. We demonstrate that *NEK8* mutations lead to defective Hippo pathway regulation, with a decreased amount of nuclear YAP in proliferating cells (missense mutations) as well as maintenance of a pool of nuclear YAP in confluent ciliated cells (missense and loss-of-function mutations) *in vitro* in patient fibroblasts. Such defects were also observed in shNEK8 mIMCD3 spheroids and *in vivo* in *Jck* cystic tubular cells. Maintenance of nuclear YAP in patient confluent ciliated cells was accompanied by higher expression of YAP targets compared to controls, sustained Ki-67 staining and abnormal cell cycle with an increased proportion of cells in G2/M. Therefore, NEK8 mutant cells still undergo proliferation and fail to differentiate when reaching confluence. Moreover, we show that cells harboring NEK8 missense mutations are more subject to DNA damage than NEK8 defective cells and undergo apoptosis, which can contribute to the difference in cell growth resulting in hypodysplastic *versus* enlarged multicystic kidneys respectively (S9 Fig).

Several mechanisms could account for nuclear YAP misregulation. Association of NEK8 with TAZ, the other major Hippo pathway effector, favors TAZ translocation into the nucleus [6, 15]. We show that NEK8 interacts with YAP in a perinuclear region, suggesting a similar regulation process for YAP and TAZ. Moreover, we show that NEK8 missense mutations alter YAP nuclear translocation. As all mutations in both kinase and RCC1 domains affect NEK8 localization into the nucleus in mIMCD3 cells (S3E and S3E’ Fig), NEK8 mutant forms might thus affect YAP shuttling into the nucleus, through their own defective nuclear translocation. NPHP4 has previously been reported to favor NEK8 and TAZ translocation into the nucleus [15]. We can hypothesize that NEK8 mutations affect binding to NPHP4, resulting in a less efficient translocation of NEK8, and consequently YAP into the nucleus. During normal cell differentiation, ciliogenesis and quiescence are accompanied by activation of the Hippo pathway (i.e. YAP inactivation) and proteasome-mediated degradation of cytoplasmic NEK8 [24]. However, the constant level of NEK8 protein expression detected in low and high confluent cells of PT1 with missense mutations suggests that these mutations preserve NEK8 from degradation, resulting in maintenance of nuclear YAP and consequently the lack of proper cell differentiation. This result is in agreement with our *in vivo* observations showing that NEK8 overexpression in zebrafish embryos mimics the YAP overexpression phenotype [38]. Moreover, the preserved YAP nuclear localization in cells with NEK8 loss-of-function mutations indicates that other proteins help YAP to translocate into the nucleus in proliferative condition, but also control its partial downregulation in confluent cells. This may explain why loss-of-function NEK8 mutations partially preserve nephrogenic differentiation, evidenced by the presence of some mature glomeruli in fetal renal biopsies.

The maintenance of YAP in the nucleus in confluent patient fibroblasts and renal cells in 3D culture may also be associated with defective Hippo pathway activation at the cilium in NEK8 mutant conditions. Recent studies on MST1/2, two major activators of the Hippo pathway, showed that they localize at the basal body and promote ciliogenesis [28]. In this study, we report the presence of phospho-YAP at the cilium and that this localization is partially affected in the presence of NEK8 missense mutations (S9 Fig). Phospho-YAP at the cilium may thus be a key component of activation of Hippo pathway under the control of NEK8. Finally, soft matrix, such as matrigel in 3D culture assays, is known to promote cytoplasmic retention of YAP and TAZ resulting in limited cell growth, *via* LATS independent non-canonical Hippo pathway activation, involving cytoskeleton, cell junctions, RhoGTPases or GPCR signaling [40]. Maintenance of nuclear YAP in enlarged spheroids in the absence of NEK8 suggests that NEK8 could also regulate YAP through non-canonical mechanisms.

The Hippo pathway is a highly integrative pathway whose regulation is connected to that of many other signalings crucial for organogenesis, including Wnt/β-catenin, TGF-β, BMP and Notch. YAP dysregulation due to NEK8 mutations is thus expected to play a major role in the development of the multisystemic defects presented by the patients/fetuses. Specific inactivation of Yap in the nephrogenic lineage (*Yap*^CM-/-^) leads to a reduced number of nephrons [41], as seen in the patients with *NEK8* missense mutations. We can hypothesize that an abnormal amount of nuclear YAP during kidney development, as shown in patient fibroblasts and *Nek8*^jck/jck^ mice, would affect the expression of downstream targets that might participate to the pathophysiological processes. *CYR61*, a gene expressed in tubules and glomeruli in fetal kidney, encodes a CCN protein that interacts with integrins to mediate cell adhesion, migration and differentiation during nephrogenesis. Upregulation of the Notch1 ligand JAG1 may induce a dysregulation of Notch pathway, required during nephron tubular development [42, 43], thus contributing to renal hypodysplasia. Finally, *CTGF*, encoding a regulator of cartilage morphogenesis and mediator of fibrosis [44, 45] could be involved in the formation of cartilage islets and fibrosis observed on most of the kidney biopsies of *NEK8* mutated patients/fetuses. In the liver, YAP is highly expressed in bile ducts and regulates the Notch pathway for ductal specification during development [31]. In the pancreas, YAP-TEAD regulates the transcriptional network controlling pancreatic cell proliferation and differentiation [46]. As for kidney defects, liver and pancreas defects vary according of the type of *NEK8* mutation, suggesting that NEK8 loss of function and gain of function differentially affect the hepatic/pancreatic transcriptional program, leading to either proliferation or paucity of bile ducts in the liver, and cysts or fibrosis in the pancreas, respectively. Finally, Hippo pathway dysregulation is associated with heart overgrowth in mice [47], thus highlighting the involvement of this pathway in the pathophysiological mechanisms leading to cardiac defects seen in the *NEK8* patients/fetuses.

In conclusion, we demonstrate that NEK8 is a multifunctional protein whose alterations lead to severe developmental abnormalities due to the synergic effect of dysfunction of key processes and signaling pathways. The demonstration of the central role of YAP dysregulation in NEK8 mutant conditions highlights potential therapeutic targets for the patients.

## Materials and Methods

### Ethics statement

This study was conducted with the approval of the « Comité de Protection des Personnes pour la Recherche Biomédicale Ile de France II ». Approval was obtained under numbers 2007-02-09/DC-2008-229 and 2009-164/DC-2011-1449 (fetuses) and 2008-A01039-46/DC-2008-229 (nephronophthisis patients). For each patient/fetus, written informed consent was obtained from the parents. For studies using animal data: housing and handling of mice were performed in accordance with the guidelines established by the French Council on animal care "Guide for the Care and Use of Laboratory Animals": EEC86/609 Council Directive—Decree 2001–131. The project was approved by the departmental director of "Services Vétérinaires de la Préfecture de Police de Paris" and by the ethical committee of the Paris Descartes University (approval number: A75-15-34).

### Patients

342 patients with isolated or syndromic NPH and 200 fetuses or early neonatal death cases with syndromic cystic dysplasia, including Meckel and Ivemark syndromes, were studied. Genomic DNA was isolated from peripheral blood or frozen tissues using standard procedures.

### *NEK8/NPHP9* mutation screening using “Ciliome” sequencing

Ciliary exome targeted sequencing and bioinformatic filtering was conducted in affected individuals using a custom SureSelect capture kit (Agilent Technologies) targeting 4.5 Mb of 20,168 exons (1221 ciliary candidate genes), including *NEK/NPHP9*. Briefly, Agilent SureSelect capture libraries were prepared from 3 μg of genomic DNA samples sheared with a Covaris S2 Ultrasonicator according to manufacturer’s instructions. The SOLiD molecular barcodes for traceable ID of samples were added at the end of the capture step. The Ovation Ultralow System (NuGEN Technologies) was used to prepare HiSeq2500 pre-capture barcoded libraries. The ciliome capture by hybridization was performed on a pool of 10 to 16 barcoded precapture libraries. Sequencing performed on SOLiD5500XL (Life Technologies) and HiSeq2500 (Illumina) was done on pools of barcoded ciliome librairies (64 barcoded ciliome libraries per SOLiD FlowChip and 16 ciliome libraries per lane of HiSeq FlowCell). Paired-end reads were generated (75 + 35 base reads for SOLiD, 100 + 100 base reads for HiSeq) and mapped on human genome reference (NCBI build37/hg19 version) using Burrows-Wheeler Aligner (Illumina) or mapread (SoliD). Downstream processing was carried out with the Genome Analysis Toolkit (GATK), SAMtools, and Picard Tools, following documented best practices (http://www.broadinstitute.org/gatk/guide/topic?name=best-practices). All variants were annotated using a software system developed by the Paris Descartes University Bioinformatics platform. The mean depth of coverage obtained was greater than 90x, and more than 89% of the exome was covered at least 15x. Different filters were applied to exclude all variants located in non-exonic regions, pseudogenes, UTRs or known polymorphic variants with a frequency above 1%, i.e. present in databases such as dbSNP, 1000 genome projects and all variants identified by in-house exome sequencing (5150 exomes and 1020 ciliomes). The functional consequence of missense variants was predicted using SIFT (http://sift.jcvi.org/www/SIFT_enst_submit.html) and PolyPhen2 (http://genetics.bwh.harvard.edu/pph2/) softwares.

### Human primary fibroblasts, mIMCD3 and HEK293T cell culture

Control and affected individual fibroblasts were cultured in Opti-MEM supplemented with 10% fetal bovine serum, penicillin, streptomycin, uridine, sodium pyruvate and Ultroser G G serum substitute (Pall Corporation). Control and patient fibroblasts (1.5 × 10^4^ or 2.5× 10^4^ cells respectively) were plated on coverslips and grown for 2 days (low confluence) or 6 days followed by 48-hour serum deprivation (high confluence) before fixation. Murine inner medullary collecting duct (mIMCD3) cells were cultured in DMEM F-12 and HEK293T in DMEM both supplemented with 10% fetal bovine serum, penicillin, streptomycin and L-Glutamine (all from Life Technologies). For immunofluorescence, 2.5 x 10^4^ cells were plated on coverslips and grown for 5 days before fixation. In all the experiments, the level of confluence was visually checked and counted to ensure similarity between control and samples.

### Immunofluorescence staining

mIMCD3 and fibroblasts were fixed in 4% PFA in PBS 1X for 15 min followed by treatment with 50 mM NH_4_Cl for 15 min. Antibodies used for immunofluorescence were: NEK8 (kind gift of D. Beier [32]), ANKS6 (1:50, Sigma-Aldrich HPA008355), GFP rabbit (1:500, Life Technology A11122), GFP chicken (2B Scientific, 1020), GM130 (1:50, BD 558712), YAP (1:50, Cell Signaling #4911), phospho-YAP (Ser127) (1:50, Cell Signaling #4911), γH2AX (1:500, Millipore 05–636) and anti-acetylated α-tubulin (1:10000, Sigma-Aldrich). Cells were permeabilized with Triton 0.5% for 10 min at room temperature and treated with blocking solution constituted of PBS 1X, 0.1% Tween 20, 3% (for fibroblasts) or 1% (for mIMCD3) BSA before incubating with primary antibodies overnight. Then, cells were washed 3 times with PBS 1X for 10 min and stained with appropriate Alexa Fluor-conjugated secondary antibodies (1:200, Molecular Probes). Nuclei were stained with Hoechst. For Annexin-V assays, cells were first incubated with a cold solution constituted by 10 mM HEPES, 140 mM NaCl and 25 mM CaCl_2_. Annexin V (Life Technologies) was secondly incubated in the same solution for 30 minutes at room temperature. Fixation was performed with PFA (4%) for 20 minutes and nuclei were stained with Hoechst.

Tissue biopsies embedded in paraffin blocks were sectioned (8 μm section thickness) using a Leica microtome. Next, sections were immersed in xylene baths (5 minutes in the first bath, 5 minutes in the second bath), then rehydrated for 5 minutes in ethanol baths of decreasing concentrations (100%, 95%, 70%, and 40%) and finally immersed in MilliQ water for 5 minutes. Dako target retrieval solution (Dako ref. S1699) was used according to the manufacturer's instructions. The slides were blocked for 45 minutes at 4°C by 10% NDS (Normal Donkey Serum) diluted in PBT (DPBS with 0.1% Triton X100). Fluorescein label Peanut Agglutin (PNA) (1:200, Vector Fl-1071) was used to detect collecting tubules. Other primary and secondary antibodies were used as described above. Slides were mounted in adapted medium, and analysed under an inverted confocal microscope Zeiss LSM 700.

### Plasmids and lentiviral infections

Nek8-knockdown (KD) was performed in mIMCD3 cells by lentiviral infection of a shRNA expressing construct in pLKO puromycin vector (Sigma-Aldrich sh1570), as previously described [39]. Puromycin-resistant Nek8-KD cells were then transfected using Lipofectamine2000 with wild-type and mutated human NEK8-GFP constructs [24] and stable cell lines re-expressing NEK8-GFP were selected with double selection with geneticin and puromycin. NEK8-GFP variants were obtained through site-directed mutagenesis using Pfu turbo kit (Invitrogen). YAP-myc construct has been described in [18].

### Coimmunoprecipitation and immunoblotting

HEK293 cells were transiently transfected using the calcium phosphate method. After 48 hours, cells were harvested with ice-cold PBS 1X. A small aliquot of this cell suspension was immediately removed and lysed directly in SDS-PAGE sample buffer as a whole cell lysate. The remaining harvested cells were lysed and treated in accordance with the Miltenyi-Biotec beads protocol. Protein dosage was performed using the BCA protein assay kit (Thermo Scientific). Fifty micrograms of proteins were loaded on a 8% acrylamide gel (Bio-rad), and Western blot was conducted using the indicated anti-FLAG M2 (1:1000, Sigma-Aldrich F1804), anti-GFP (1:1000, Roche #1814460001), anti-tubulin (1:10000, Sigma-Aldrich T5168).

### Proximity Ligation Assay

For *in situ* Proximity Ligation Assay (PLA) (OLINK Biosciences, Uppsala Sweden), HEK293 cells were fixed 48h after transfection in 4% PFA for 15 min, permeabilized 10 min with PBS-0.1% Triton before treated with blocking solution, labeled with anti-rabbit GFP and anti-mouse Myc (Thermo Fischer Scientific, #MS139P1) antibodies and then incubated with a pair of nucleotide-labeled secondary antibodies (rabbit PLA probe MINUS and mouse PLA probe PLUS) in hybridization solution. Interactions between the PLA probes, possible when within a distance less than 40 nm, were revealed by adding a ligase and by amplification of a rolling-circle product using labeled oligonucleotides and a polymerase, according to the manufacturer's instructions. Signals indicative of interactions were detected by confocal microscopy as fluorescent dots in visible red.

### RNA extraction and RT-PCR

Total cellular mRNA was isolated using Qiagen Extraction Kit and then treated with DNase I. 1.5 μg of total RNA was reverse-transcribed using Superscript II (Life Technologies). Relative expression levels of genes of interest were determined by real-time RT-PCR using the Absolute SYBR Green ROX Mix (ABgene) and specific primers as follows: human *NEK8* forward 5’-GCCTCAAGAGGGCTTTCGA-3’ and reverse 5’-AAGGTGCCACTCATGATCTTCAG-3’; mouse *Nek8* forward 5'-GCACCTTGGCCGAGTTCAT-3' and reverse 5'-GCCAGCAGGATCTGCACAA-3'; human *CTGF* forward 5'-CGAAGCTGACCTGGAAGAGAA-3' and reverse 5'- GTACTCCCAAAATCTCCAAGCCT-3'; human *CYR61* forward 5'-GAGTGGGTCTGTGACGAGGAT-3' and reverse 5'-GGTTGTATAGGATGCGAGGCT -3'; human *TEAD4* forward 5'-GGACACTACTCTTACCGCATCC-3' and reverse 5'- TCAAAGACATAGGCAATGCACA-3; human *JAG1* forward 5'-GCCGAGGTCCTATACGTTGC-3' and reverse 5'-CCGAGTGAGAAGCCTTTTCAA-3'; human *HES1* forward 5'-TCAACACGACACCGGATAAAC-3' and reverse 5'-GCCGCGAGCTATCTTTCTTCA-3'. Experiments were repeated at least three times and gene expression levels were normalized to *GAPDH*.

For qPCR analyses in IMCD3 cells, we used mouse primers described below.

### Studies on *Jck* mice

Experiments were performed on 5-week-old female mutant juvenile cystic kidney (*Jck*) mice bearing a *Nek8* mutation (The Jackson Laboratory) and compared to wild-type littermates. Animals were fed *ad libitum* and housed at constant ambient temperature in a 12/12-hour light/dark cycle.

For mouse samples, 4 μm sections of paraffin-embedded kidneys were submitted to heat-mediated antigen retrieval and incubated with antibody to Yap (Cell Signaling Technology, 4912, 1:100), followed by a donkey anti-rabbit biotinylated antibody (GE Healthcare) at 1:200. Biotinylated antibodies were detected using HRP-labeled streptavidin (Dako) at 1:2000 and 3–3′-diamino-benzidine-tetrahydrochloride (DAB) revelation.

Western blot analyses were performed as previously described [48]. Briefly, protein extracts from kidneys were resolved by SDS-PAGE before being transferred onto the appropriate membrane and incubated with antibodies to phospho-YAP (Ser127) (Cell Signaling Technology, 4911, 1:1000), and YAP (Santa Cruz, sc-101199, 1:1000), Gapdh (Millipore, 1:5000) followed by the appropriate Alexa-conjugated secondary antibody (Life Technologies). Fluorescence was acquired using a ChemiDoc MP Imaging System (Bio-Rad), and densitometry was performed using Image Lab software 5.0.

For real-time RT-PCR, mRNA were extracted from whole kidney samples and *Ctgf*, *Cyr61*, *Birc5* and *Ankrd1* expression were analysed by real-time RT-PCR using CFX96 Touch Real-Time PCR Detection System (Bio-Rad). Primers (Eurogentec) were as follows: *Ctgf* forward 5’-GCTGACCTGGAGGAAAACATTAA-3’ and reverse 5’-TGACAGGCTTGGCGATTTTAG-3’; *Cyr61* forward 5’-CCTTCTCCACTTGACCAGAC-3’ and reverse 5’-ATATTCACAGGGTCTGCCTTCT-3’; *Birc5* forward 5’-CCCGATGACAACCCGATAGAG-3’ and reverse 5’-TGACGGGTAGTCTTTGCAGTC-3’; *Ankrd1* forward 5’-CTGTGAGGCTGAACCGCTAT-3’ and reverse 5’-CCAGTGCAACACCAGATCCA-3’. *Rpl13* was used as the normalization control.

### Cell growth rate assay and cell cycle analysis

A total of 7.5 x 10^4^ cells/well were plated in triplicate in 6 well plates and grown for 1–7 days. Cells were incubated with complete medium as previously described. The number of cells was counted at the indicated time-points in triplicate.

For flow cytometry analysis, cells were plated at a density of 1 x 10^5^ cells/ml. The cells were pulse-labeled with BrdU for 30 min, washed with PBS, and treated with trypsin. Cells were fixed with ethanol and stained with anti-BrdU-FITC antibody (BD Biosciences) and propidium iodide, according to the manufacturer’s instructions. Flow cytometry analysis was carried out with the BD LSRII flow cytometry system and BD FACSDiva software.

### mIMCD3 3D culture in matrigel and immunofluorescence

96 well plates were coated with a thin layer of collagen (collagen I, Rat Tail, Corning #354236) that was allowed to polymerize at 37° C for 30 minutes. 4 x 10^4^ cells per well in the appropriate medium (with antibiotics) was mixed with Matrigel (BD) and allowed to polymerize at 37°C for 30 minutes. Subsequently, the appropriate medium was added and changed every 2 days. Samples were fixed after 2, 3 or 5 days of culture. After two washes with PBS 1X, PFA 4% was added for 30 minutes. Antibody stainings were done as previously described; only incubation time with blocking solution was prolonged to 1 hour at room temperature.

### Zebrafish experiments

Zebrafish were maintained at 28.5°C under standard protocols. *Tg(cmlc2*:*GFP)* and *Tg(wt1b*:*GFP)* transgenic lines were used to assess heart looping and pronephros morphology, respectively. Control and *nek8* (ATG) morpholinos [37] were injected into one-cell stage embryos at 0.4 pmol per embryo. Human full length *NEK8-GFP* RNA was obtained by *in vitro* transcription with mMESSAGE mMACHINE kit (Ambion) and injected into one-cell stage embryos at 100 pg per embryo. For Verteporfin treatment, embryos were injected with 100 pg of RNA and GFP-positive animals were selected at shield stage. Embryos were then treated with either DMSO or 20 μM Verteporfin from 90% epiboly stage to 34 or 52 hours post fertilization (hpf), time points at which body curvature and laterality/pronephros phenotypes were measured, respectively. Phenotypes were analysed using a Leica M165FC stereoscope.

For real-time RT-PCR, mRNA was extracted from whole embryos at 34 hpf by TRIZOL and *ctgfa*, reported to be specific to Yap unlike *ctgfb* [49], c*yr61* and *tead4* expression were analysed by real-time RT-PCR using Absolute SYBR Green ROX Mix (ABgene) and specific primers as follows: c*tgfa* forward 5’-TCCTCACAGAACCGCCACCTTGCCCAT-3’ and reverse 5’-TCACGCCATGTCGCCAACCATCTTCTTGT-3’; *cyr61* forward 5’-CCGTGTCCACATGTACATGGG-3’ and reverse 5’-GGTGCATGAAAGAAGCTCGTC-3’; *tead4* forward 5’-AAGGAGGACTGAAGGAGCTGTTCGAGAAGG-3’ and reverse 5’-GCCGAATGAGCAGACTTTAGTGGAGGAGGT-3’. *gapdh* was used as the normalization control.

### Verteporfin treatment

For treatment of human fibroblasts, Verteporfin (Sigma-Aldrich, SML0534) was added after 5 days of culture when cells had achieved confluence. Several drug concentrations were tested and 0.5–0.075 μM were chosen as optimal non toxical conditions. For treatment of shNEK8 re-expressing NEK8-GFP mIMCD3 cells, we used drug concentration ranging from 0.5 to 4 μM. Verteporfin treatment was also used on control pLKO1 and shNEK8 mIMCD3 cells for rescue experiments in the matrigel 3D assay. In this case, the drug (1 and 2 μM) was added after 2 days of culture and maintained until fixation at 3 or 5 days.

## Supporting Information

S1 Fig Human *NEK8* mutations. (A) Chromatograms of the eight different *NEK8* mutations identified in individuals with renal cystic hypodysplasia and associated defects. Family numbers and predicted translational changes are indicated. Sequence traces are shown for mutations above normal controls or heterozygous carriers. Arrowheads indicate mutated nucleotides. (B) Quantification of the level of expression of *NEK8* in fibroblasts from affected cases of families 1 and 5 (PT1 and PT5) showing partial RNA decay in PT5 cells.
(TIF)

S2 Fig T87A/R602W NEK8 mutations lead to persistent localization of NEK8 at the Golgi membranes. (A) Serum-starved control and patient fibroblasts stained for NEK8 (red) and the Golgi marker, GM130 (green). (B) Control fibroblasts transiently transfected with WT NEK8-GFP and GFP constructs were fixed after 48 hours and labeled for GFP (green) and GM130 (red). Images show the co-localization of WT NEK8-GFP at the Golgi membranes. (C) Low density control fibroblasts staining for NEK8 (red) and the Golgi marker, GM130 (green). Scale bar, 10 μm.
(TIF)

S3 Fig Establishment of *Nek8* depleted mIMCD3 cell line (shNEK8) re-expressing NEK8-GFP WT and mutants. (A) Murine *Nek8* mRNA levels were analyzed by qPCR in mIMCD3 (mIMCD3 WT), control pLKO and shNEK8 cells. (B) Nek8 extinction was also analyzed by immunostaining. Staining of NEK8 (red), acetylated α-tubulin (green) and nuclei (Hoechst, blue) were performed in control pLKO and shNEK8 cells. Scale bar, 10 μm. (B’) Quantification of NEK8 positive cilia in shNEK8 cells. **p < 0.01, calculated by Student *t*-*test* with Welsh correction. (C, D) analysis of the expression of human *NEK8* in the shNEK8 cell re-expressing WT and mutant NEK8-GFP by qPCR (C) and western blot (D). (E) Nuclear localization of GFP-NEK8 (green) in mIMCD3 cells transfected with plasmids encoding GFP-tagged NEK8 wild type (WT) or patients' variants. Stack images of the nucleus are shown. Scale bar, 10 μm. (E’) Ratio of the GFP intensity in the nucleus *versus* cytosol, showing that NEK8 mutations affect its nuclear localization. *p < 0.05, **p < 0.01, ***p < 0.001, calculated by Bonferroni post-hoc test following ANOVA.
(TIF)

S4 Fig NEK8 mutations alter cell cycle progression in fibroblasts. (A) Cell cycle analysis by flow cytometry of control and patient fibroblasts cultivated in low (top) and high cell density followed for 48 hours of serum starvation (bottom). Cells in S-phase stage were labeled with BrdU and DNA content was determined by propidium iodide staining. (A’) Table presenting the average percentage of cells in each phase of cell cycle, in low (top) and high (bottom) cell density conditions.
(TIF)

S5 Fig *NEK8* mutations do not affect YAP phosphorylation on Serine 127. (A) Control and patient fibroblasts were fixed after 2 days (low cell density) or 6 days of culture in standard medium followed by 2 days of serum starvation (high cell density). Cells were stained with anti phospho-YAP antibody (red) and nuclei (Hoechst, blue). Scale bar, 10 μm. (A’) Quantification of phospho-YAP staining. *p < 0.05, **p < 0.01, calculated by Kruskall-Wallis test.
(TIF)

S6 Fig Decreased nuclear YAP localization in presence of missense mutated NEK8 proteins and NEK8/YAP interaction in co-transfected HEK293 cells. (A) HEK293T cells were co-transfected with WT or mutated NEK8-GFP and YAP-MYC constructs, fixed after 48 hours and stained for GFP (green) and MYC (red). Scale bar, 10 μm. (A’) Graph representing the ratio between nuclear and cytosolic YAP intensities, based on three independent experiments. ** p < 0.01, *** p < 0,001, calculated via Bonferroni post-hoc tests following ANOVA. (B) 48h after transfection, cells were fixed and a proximity ligation assay was performed using the appropriate anti-GFP and anti-myc antibodies, showing that YAP and NEK8 WT are in close vicinity. Scale bar, 10 μm.
(TIF)

S7 Fig Efficiency of Verteporfin treatment on YAP target gene expression in mIMCD3 and fibroblast cells. qPCR analyses of YAP target gene expression in DMSO- and Verteporfin (VP)-treated control (pLKO) and shNEK8 mIMCD3 cells (A), as well as in control and patient (PT1) fibroblasts (B). In both cell lines, NEK8 mutations lead to upregulation of YAP target genes, which is blocked upon Verteporfin treatment.
(TIF)

S8 Fig Verteporfin treatment partially rescues pronephric defects induced by NEK8 overexpression in zebrafish embryos. (A) Representative images of body axis, laterality (heart looping) and pronephros defects observed in zebrafish embryos. Four classes have been determined depending of the body shape, class I (blue) for normal embryos, class II (orange) for embryos with shortened axis, class III (red) for embryos with severely shortened and dorsally curved body axis, and class IV (black, only observed with *nek8* MO) with ventrally curved body axis. Laterality defects encompass no looped and right-sided hearts compare to normal left-sided heart. Ventral views, anterior to the top. Pronephros defects encompass cystic glomeruli (asterisks) and developmental (Dvlpt) abnormalities. Dorsal views, anterior to the top. *Tg(cmlc2*:*GFP)* and *Tg(wt1b*:*GFP)* transgenic lines were used to observe heart looping and pronephros morphology, respectively. (A’) Graphs representing the proportions of embryos presenting laterality defects (top panel) and pronephric cysts (bottom panel) in control MO-, *nek8* MO- and *nek8* MO/human *NEK8-GFP* RNA-injected embryos. (A”) Graphs representing the proportions of embryos presenting laterality (left panel) and pronephros (right panel) defects (dashed bars) within each class of body axis shape, in control *GFP* and human *NEK8-GFP* RNA-injected embryos, treated with DMSO or Verteporfin (VP, 20 μM) from 90% epiboly to 34 hpf. (B) qPCR analysis of Yap target gene expression, *tead4*, *ctgfa*, *cyr61*, in human *NEK8-GFP* RNA-injected embryos treated with DMSO or Verteporfin (VP, 20 μM) compare to control *GFP* RNA injected embryos.
(TIF)

S9 Fig Schematic model of ciliary and YAP regulation mediated by NEK8 in control and in patient cells. **In control cells** at low cell density, NEK8 participates by its nuclear localization in insuring an efficient YAP-dependent transcriptional activity, allowing proliferation and cell survival. At high cell density, the Hippo pathway is activated and the total amount of NEK8 protein decreases through proteasomal degradation. We suggest that a decrease of NEK8 protein level facilitates the degradation of cytosolic YAP, stopping transcription of target genes. In parallel, NEK8 is targeted at the INVS compartment into the primary cilium, promoting the recruitment of both phospho-YAP and ANKS6 at the cilia. Altogether, cellular signals converge in the inhibition of proliferation in favour of differentiation. In patient cells at low cell density, ***NEK8* missense mutations** prevent the nuclear localization of NEK8, thus causing a reduction of nuclear YAP localization and activity. As a consequence, patient cells fail to proliferate as much as control cells while undergoing both apoptosis and genomic instability. We hypothesize that *NEK8* missense mutations also affect NEK8 proteasomal degradation, which participates in the maintenance of YAP into the nucleus and a sustained low level of transcriptional activity/proliferation when cells reach confluence. In parallel, missense mutations induce ciliogenesis defects and prevent NEK8 to localize at the cilium, resulting in defective INVS compartment integrity and loss of phospho-YAP at the cilium. Consequently, induction of differentiation is severely altered. In presence of **NEK8 loss-of-function mutation**s, YAP nuclear translocation occurs under the control of (an)other, unidentified factor(s), resulting in a high level of proliferation. At high cell density, absence of NEK8, mimicking degradation of the protein that normally occurs, allows a reduction of the pool of nuclear YAP. However, as in the context of missense mutations, some YAP remains in the nucleus and proliferation is not arrested. Absence of NEK8 does not prevent cilium formation nor ciliary localization of phospho-YAP, but the INVS compartment is defective. Consequently, differentiation partially occurs but the balance between proliferation and differentiation is altered.
(PPTX)

S10 Fig *ANKS6* loss of function causes a similar phenotype than *NEK8* mutations in human. (A-B) Pedigree (A) and chromatograms (B) of family 6 identified with *ANKS6/NPHP16* mutation. The mutation is numbered according to the human cDNA (NM_173551). Position +1 corresponds to the A of ATG. Abbreviations in the table are: CS, consanguinity; **Termination of pregnancy at 28 weeks of gestation.
(TIF)

## References

[1] Benzing T, Schermer B. Clinical spectrum and pathogenesis of nephronophthisis. Current opinion in nephrology and hypertension. 2012;21(3):272–8. 10.1097/MNH.0b013e3283520f17 . 22388554

[2] Wolf MT. Nephronophthisis and related syndromes. Current opinion in pediatrics. 2015;27(2):201–11. 10.1097/MOP.0000000000000194 25635582 PMC4422489

[3] Fischer E, Legue E, Doyen A, Nato F, Nicolas JF, Torres V, et al Defective planar cell polarity in polycystic kidney disease. Nature genetics. 2006;38(1):21–3. 10.1038/ng1701 . 16341222

[4] Arts HH, Knoers NV. Current insights into renal ciliopathies: what can genetics teach us? Pediatric nephrology. 2013;28(6):863–74. 10.1007/s00467-012-2259-9 22829176 PMC3631122

[5] Otto EA, Trapp ML, Schultheiss UT, Helou J, Quarmby LM, Hildebrandt F. NEK8 mutations affect ciliary and centrosomal localization and may cause nephronophthisis. Journal of the American Society of Nephrology: JASN. 2008;19(3):587–92. 10.1681/ASN.2007040490 18199800 PMC2391043

[6] Frank V, Habbig S, Bartram MP, Eisenberger T, Veenstra-Knol HE, Decker C, et al Mutations in NEK8 link multiple organ dysplasia with altered Hippo signalling and increased c-MYC expression. Human molecular genetics. 2013;22(11):2177–85. 10.1093/hmg/ddt070 . 23418306

[7] Fry AM, O'Regan L, Sabir SR, Bayliss R. Cell cycle regulation by the NEK family of protein kinases. Journal of cell science. 2012;125(Pt 19):4423–33. 10.1242/jcs.111195 23132929 PMC3500863

[8] Shiba D, Manning DK, Koga H, Beier DR, Yokoyama T. Inv acts as a molecular anchor for Nphp3 and Nek8 in the proximal segment of primary cilia. Cytoskeleton. 2010;67(2):112–9. 10.1002/cm.20428 . 20169535 PMC9134828

[9] Bergmann C, Fliegauf M, Bruchle NO, Frank V, Olbrich H, Kirschner J, et al Loss of nephrocystin-3 function can cause embryonic lethality, Meckel-Gruber-like syndrome, situs inversus, and renal-hepatic-pancreatic dysplasia. American journal of human genetics. 2008;82(4):959–70. 10.1016/j.ajhg.2008.02.017 18371931 PMC2427297

[10] Czarnecki PG, Gabriel GC, Manning DK, Sergeev M, Lemke K, Klena NT, et al ANKS6 is the critical activator of NEK8 kinase in embryonic situs determination and organ patterning. Nature communications. 2015;6:6023 10.1038/ncomms7023 25599650 PMC4361001

[11] Hoff S, Halbritter J, Epting D, Frank V, Nguyen TM, van Reeuwijk J, et al ANKS6 is a central component of a nephronophthisis module linking NEK8 to INVS and NPHP3. Nature genetics. 2013;45(8):951–6. 10.1038/ng.2681 23793029 PMC3786259

[12] Okada M, Sugimoto K, Shimada Y, Fujita S, Yanagida H, Yagi K, et al Association of INVS (NPHP2) mutation in an adolescent exhibiting nephronophthisis (NPH) and complete situs inversus. Clinical nephrology. 2008;69(2):135–41. . 18218308 10.5414/cnp69135

[13] Tory K, Rousset-Rouviere C, Gubler MC, Moriniere V, Pawtowski A, Becker C, et al Mutations of NPHP2 and NPHP3 in infantile nephronophthisis. Kidney international. 2009;75(8):839–47. 10.1038/ki.2008.662 . 19177160

[14] Choi HJ, Lin JR, Vannier JB, Slaats GG, Kile AC, Paulsen RD, et al NEK8 links the ATR-regulated replication stress response and S phase CDK activity to renal ciliopathies. Molecular cell. 2013;51(4):423–39. 10.1016/j.molcel.2013.08.006 23973373 PMC3790667

[15] Habbig S, Bartram MP, Sagmuller JG, Griessmann A, Franke M, Muller RU, et al The ciliopathy disease protein NPHP9 promotes nuclear delivery and activation of the oncogenic transcriptional regulator TAZ. Human molecular genetics. 2012;21(26):5528–38. 10.1093/hmg/dds408 . 23026745

[16] Varelas X. The Hippo pathway effectors TAZ and YAP in development, homeostasis and disease. Development. 2014;141(8):1614–26. 10.1242/dev.102376 . 24715453

[17] Yu FX, Guan KL. The Hippo pathway: regulators and regulations. Genes & development. 2013;27(4):355–71. 10.1101/gad.210773.112 23431053 PMC3589553

[18] Zhao B, Wei X, Li W, Udan RS, Yang Q, Kim J, et al Inactivation of YAP oncoprotein by the Hippo pathway is involved in cell contact inhibition and tissue growth control. Genes & development. 2007;21(21):2747–61. 10.1101/gad.1602907 17974916 PMC2045129

[19] Habbig S, Bartram MP, Muller RU, Schwarz R, Andriopoulos N, Chen S, et al NPHP4, a cilia-associated protein, negatively regulates the Hippo pathway. The Journal of cell biology. 2011;193(4):633–42. 10.1083/jcb.201009069 21555462 PMC3166863

[20] Failler M, Gee HY, Krug P, Joo K, Halbritter J, Belkacem L, et al Mutations of CEP83 cause infantile nephronophthisis and intellectual disability. American journal of human genetics. 2014;94(6):905–14. 10.1016/j.ajhg.2014.05.002 24882706 PMC4121475

[21] Halbritter J, Bizet AA, Schmidts M, Porath JD, Braun DA, Gee HY, et al Defects in the IFT-B component IFT172 cause Jeune and Mainzer-Saldino syndromes in humans. American journal of human genetics. 2013;93(5):915–25. 10.1016/j.ajhg.2013.09.012 24140113 PMC3824130

[22] Thomas S, Wright KJ, Le Corre S, Micalizzi A, Romani M, Abhyankar A, et al A homozygous PDE6D mutation in Joubert syndrome impairs targeting of farnesylated INPP5E protein to the primary cilium. Human mutation. 2014;35(1):137–46. 10.1002/humu.22470 24166846 PMC3946372

[23] Alessandri JL, Cartault F, Brayer C, Cuillier F, Riviere JP, Ramful D, et al Renal cystic dysplasia, paucity of bile ducts, situs inversus, bowing of the femora in two siblings in the Reunion Island: a ciliopathy? Clinical dysmorphology. 2009;18(4):209–12. 10.1097/MCD.0b013e32832b1376 . 19550299

[24] Zalli D, Bayliss R, Fry AM. The Nek8 protein kinase, mutated in the human cystic kidney disease nephronophthisis, is both activated and degraded during ciliogenesis. Human molecular genetics. 2012;21(5):1155–71. 10.1093/hmg/ddr544 22106379 PMC3277313

[25] Kuo LJ, Yang LX. Gamma-H2AX—a novel biomarker for DNA double-strand breaks. In vivo. 2008;22(3):305–9. . 18610740

[26] Giles RH, Ajzenberg H, Jackson PK. 3D spheroid model of mIMCD3 cells for studying ciliopathies and renal epithelial disorders. Nature protocols. 2014;9(12):2725–31. 10.1038/nprot.2014.181 . 25356583

[27] Varelas X, Samavarchi-Tehrani P, Narimatsu M, Weiss A, Cockburn K, Larsen BG, et al The Crumbs complex couples cell density sensing to Hippo-dependent control of the TGF-beta-SMAD pathway. Developmental cell. 2010;19(6):831–44. 10.1016/j.devcel.2010.11.012 . 21145499

[28] Kim J, Jo H, Hong H, Kim MH, Kim JM, Lee JK, et al Actin remodelling factors control ciliogenesis by regulating YAP/TAZ activity and vesicle trafficking. Nature communications. 2015;6:6781 10.1038/ncomms7781 . 25849865

[29] Pobbati AV, Hong W. Emerging roles of TEAD transcription factors and its coactivators in cancers. Cancer biology & therapy. 2013;14(5):390–8. 10.4161/cbt.23788 23380592 PMC3672182

[30] Tschaharganeh DF, Chen X, Latzko P, Malz M, Gaida MM, Felix K, et al Yes-associated protein up-regulates Jagged-1 and activates the Notch pathway in human hepatocellular carcinoma. Gastroenterology. 2013;144(7):1530–42 e12. 10.1053/j.gastro.2013.02.009 23419361 PMC3665638

[31] Yimlamai D, Christodoulou C, Galli GG, Yanger K, Pepe-Mooney B, Gurung B, et al Hippo pathway activity influences liver cell fate. Cell. 2014;157(6):1324–38. 10.1016/j.cell.2014.03.060 24906150 PMC4136468

[32] Liu S, Lu W, Obara T, Kuida S, Lehoczky J, Dewar K, et al A defect in a novel Nek-family kinase causes cystic kidney disease in the mouse and in zebrafish. Development. 2002;129(24):5839–46. . 12421721 10.1242/dev.00173

[33] Smith LA, Bukanov NO, Husson H, Russo RJ, Barry TC, Taylor AL, et al Development of polycystic kidney disease in juvenile cystic kidney mice: insights into pathogenesis, ciliary abnormalities, and common features with human disease. Journal of the American Society of Nephrology: JASN. 2006;17(10):2821–31. 10.1681/ASN.2006020136 . 16928806

[34] Archibald A, Al-Masri M, Liew-Spilger A, McCaffrey L. Atypical protein kinase C induces cell transformation by disrupting Hippo/Yap signaling. Mol Biol Cell. 2015;26(20):3578–95. 10.1091/mbc.E15-05-0265 26269582 PMC4603929

[35] Liu-Chittenden Y, Huang B, Shim JS, Chen Q, Lee SJ, Anders RA, et al Genetic and pharmacological disruption of the TEAD-YAP complex suppresses the oncogenic activity of YAP. Genes & development. 2012;26(12):1300–5. 10.1101/gad.192856.112 22677547 PMC3387657

[36] Zaghloul NA, Katsanis N. Zebrafish assays of ciliopathies. Methods in cell biology. 2011;105:257–72. 10.1016/B978-0-12-381320-6.00011–4 21951534 PMC3638959

[37] Fukui H, Shiba D, Asakawa K, Kawakami K, Yokoyama T. The ciliary protein Nek8/Nphp9 acts downstream of Inv/Nphp2 during pronephros morphogenesis and left-right establishment in zebrafish. FEBS letters. 2012;586(16):2273–9. 10.1016/j.febslet.2012.05.064 . 22687244

[38] Skouloudaki K, Puetz M, Simons M, Courbard JR, Boehlke C, Hartleben B, et al Scribble participates in Hippo signaling and is required for normal zebrafish pronephros development. Proceedings of the National Academy of Sciences of the United States of America. 2009;106(21):8579–84. 10.1073/pnas.0811691106 19439659 PMC2688978

[39] Manning DK, Sergeev M, van Heesbeen RG, Wong MD, Oh JH, Liu Y, et al Loss of the ciliary kinase Nek8 causes left-right asymmetry defects. Journal of the American Society of Nephrology: JASN. 2013;24(1):100–12. 10.1681/ASN.2012050490 23274954 PMC3537214

[40] Piccolo S, Dupont S, Cordenonsi M. The biology of YAP/TAZ: hippo signaling and beyond. Physiological reviews. 2014;94(4):1287–312. 10.1152/physrev.00005.2014 . 25287865

[41] Reginensi A, Scott RP, Gregorieff A, Bagherie-Lachidan M, Chung C, Lim DS, et al Yap- and Cdc42-dependent nephrogenesis and morphogenesis during mouse kidney development. PLoS genetics. 2013;9(3):e1003380 10.1371/journal.pgen.1003380 23555292 PMC3605093

[42] Heliot C, Desgrange A, Buisson I, Prunskaite-Hyyrylainen R, Shan J, Vainio S, et al HNF1B controls proximal-intermediate nephron segment identity in vertebrates by regulating Notch signalling components and Irx1/2. Development. 2013;140(4):873–85. 10.1242/dev.086538 . 23362348

[43] Massa F, Garbay S, Bouvier R, Sugitani Y, Noda T, Gubler MC, et al Hepatocyte nuclear factor 1beta controls nephron tubular development. Development. 2013;140(4):886–96. 10.1242/dev.086546 . 23362349

[44] Lipson KE, Wong C, Teng Y, Spong S. CTGF is a central mediator of tissue remodeling and fibrosis and its inhibition can reverse the process of fibrosis. Fibrogenesis & tissue repair. 2012;5(Suppl 1):S24 10.1186/1755-1536-5-S1-S24 23259531 PMC3368796

[45] Takigawa M. CCN2: a master regulator of the genesis of bone and cartilage. Journal of cell communication and signaling. 2013;7(3):191–201. 10.1007/s12079-013-0204-8 23794334 PMC3709051

[46] Cebola I, Rodriguez-Segui SA, Cho CH, Bessa J, Rovira M, Luengo M, et al TEAD and YAP regulate the enhancer network of human embryonic pancreatic progenitors. Nature cell biology. 2015;17(5):615–26. 10.1038/ncb3160 25915126 PMC4434585

[47] Heallen T, Zhang M, Wang J, Bonilla-Claudio M, Klysik E, Johnson RL, et al Hippo pathway inhibits Wnt signaling to restrain cardiomyocyte proliferation and heart size. Science. 2011;332(6028):458–61. 10.1126/science.1199010 21512031 PMC3133743

[48] Viau A, El Karoui K, Laouari D, Burtin M, Nguyen C, Mori K, et al Lipocalin 2 is essential for chronic kidney disease progression in mice and humans. The Journal of clinical investigation. 2010;120(11):4065–76. 10.1172/JCI42004 20921623 PMC2964970

[49] Mateus R, Lourenco R, Fang Y, Brito G, Farinho A, Valerio F, et al Control of tissue growth by Yap relies on cell density and F-actin in zebrafish fin regeneration. Development. 2015;142(16):2752–63. 10.1242/dev.119701 . 26209644 PMC6514408

