# Supplementary figures and images for: Novel NEK8 Mutations Cause Severe Syndromic Renal Cystic Dysplasia through YAP Dysregulation
Source: PLoS Genet. 2016 Mar 11;12(3):e1005894. doi: 10.1371/journal.pgen.1005894 (PMC4788435; doi:10.1371/journal.pgen.1005894)

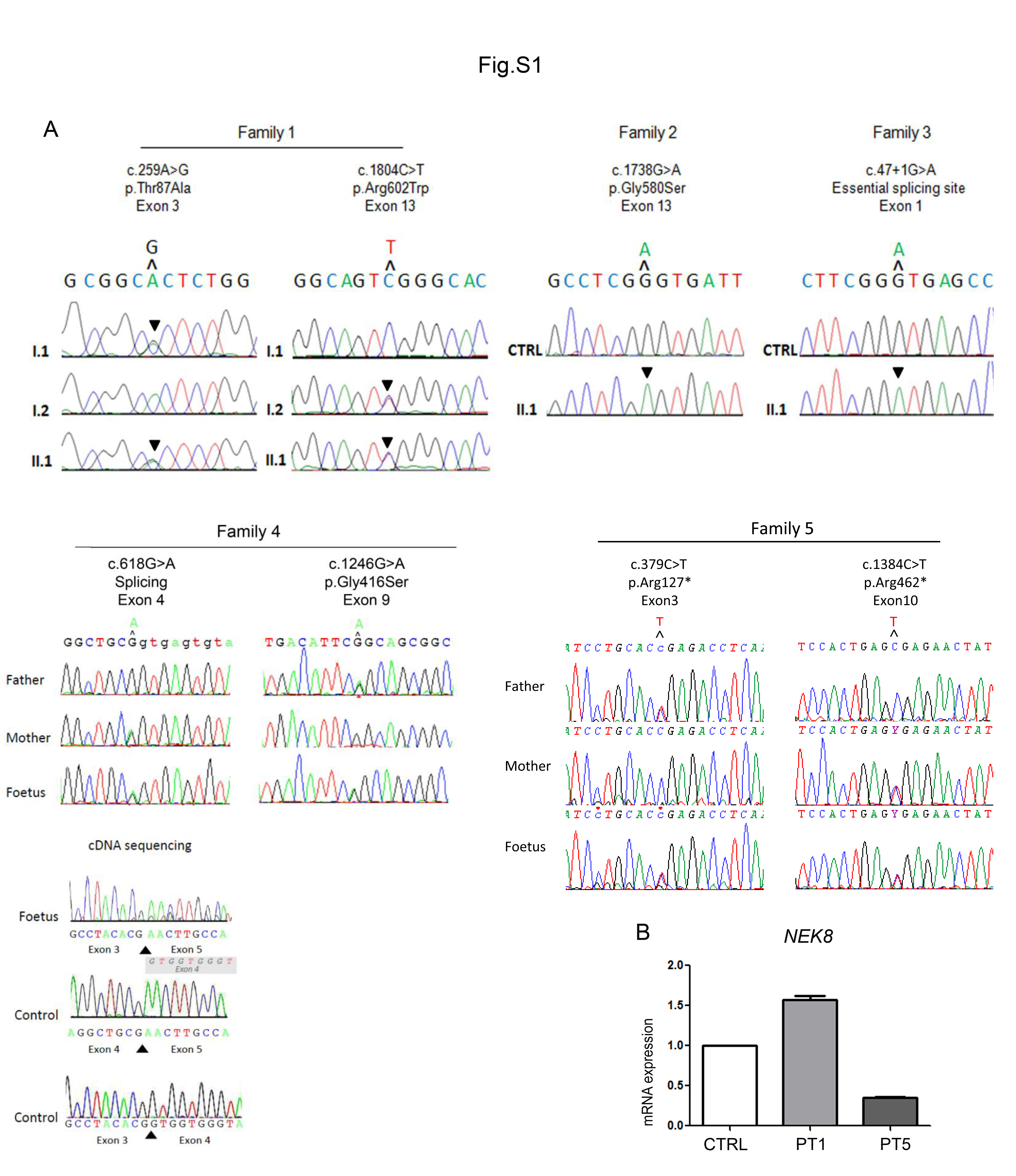

Supplement: S1 Fig — (A) Chromatograms of the eight different NEK8 mutations identified in individuals with renal cystic hypodysplasia and associated defects. Family numbers and predicted translational changes are indicated. Sequence traces are shown for mutations above normal controls or heterozygous carriers. Arrowheads indicate mutated nucleotides. (B) Quantification of the level of expression of NEK8 in fibroblasts from affected cases of families 1 and 5 (PT1 and PT5) showing partial RNA decay in PT5 cells. (TIF) [file pgen.1005894.s001.tif]

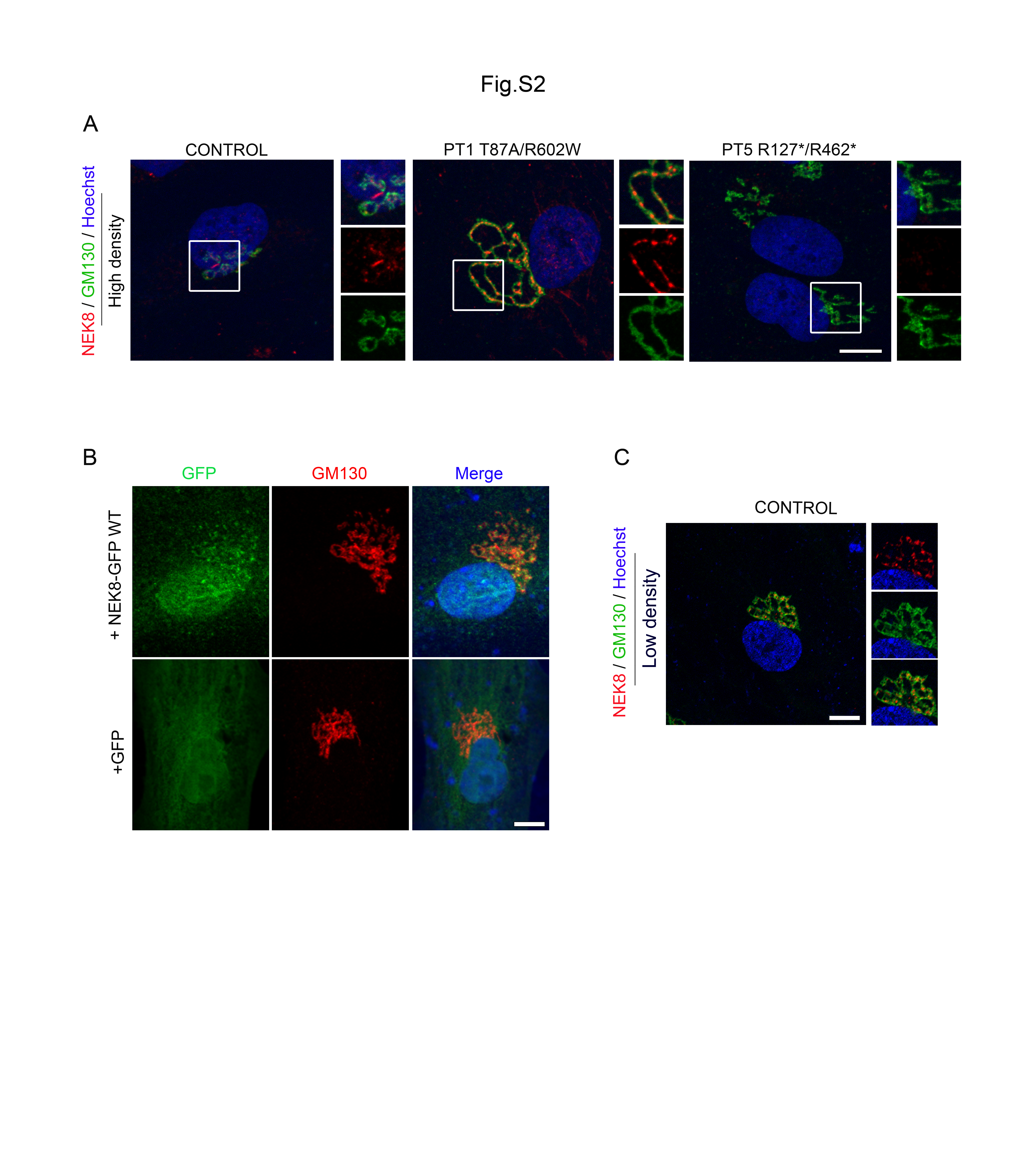

Supplement: S2 Fig — (A) Serum-starved control and patient fibroblasts stained for NEK8 (red) and the Golgi marker, GM130 (green). (B) Control fibroblasts transiently transfected with WT NEK8-GFP and GFP constructs were fixed after 48 hours and labeled for GFP (green) and GM130 (red). Images show the co-localization of WT NEK8-GFP at the Golgi membranes. (C) Low density control fibroblasts staining for NEK8 (red) and the Golgi marker, GM130 (green). Scale bar, 10 μm. (TIF) [file pgen.1005894.s002.tif]

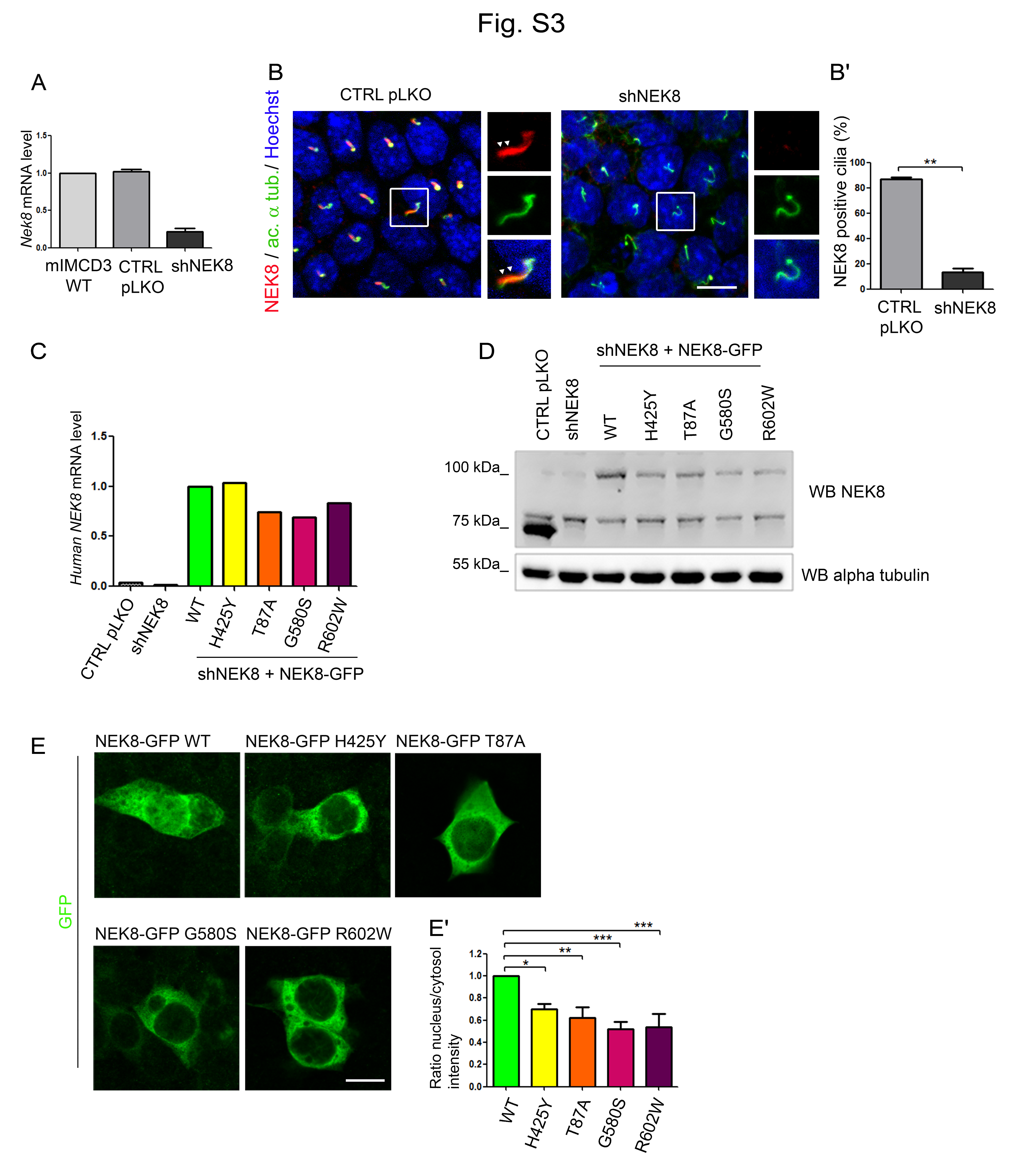

Supplement: S3 Fig — (A) Murine Nek8 mRNA levels were analyzed by qPCR in mIMCD3 (mIMCD3 WT), control pLKO and shNEK8 cells. (B) Nek8 extinction was also analyzed by immunostaining. Staining of NEK8 (red), acetylated α-tubulin (green) and nuclei (Hoechst, blue) were performed in control pLKO and shNEK8 cells. Scale bar, 10 μm. (B’) Quantification of NEK8 positive cilia in shNEK8 cells. **p < 0.01, calculated by Student t-test with Welsh correction. (C, D) analysis of the expression of human NEK8 in the shNEK8 cell re-expressing WT and mutant NEK8-GFP by qPCR (C) and western blot (D). (E) Nuclear localization of GFP-NEK8 (green) in mIMCD3 cells transfected with plasmids encoding GFP-tagged NEK8 wild type (WT) or patients' variants. Stack images of the nucleus are shown. Scale bar, 10 μm. (E’) Ratio of the GFP intensity in the nucleus versus cytosol, showing that NEK8 mutations affect its nuclear localization. *p < 0.05, **p < 0.01, ***p < 0.001, calculated by Bonferroni post-hoc test following ANOVA. (TIF) [file pgen.1005894.s003.tif]

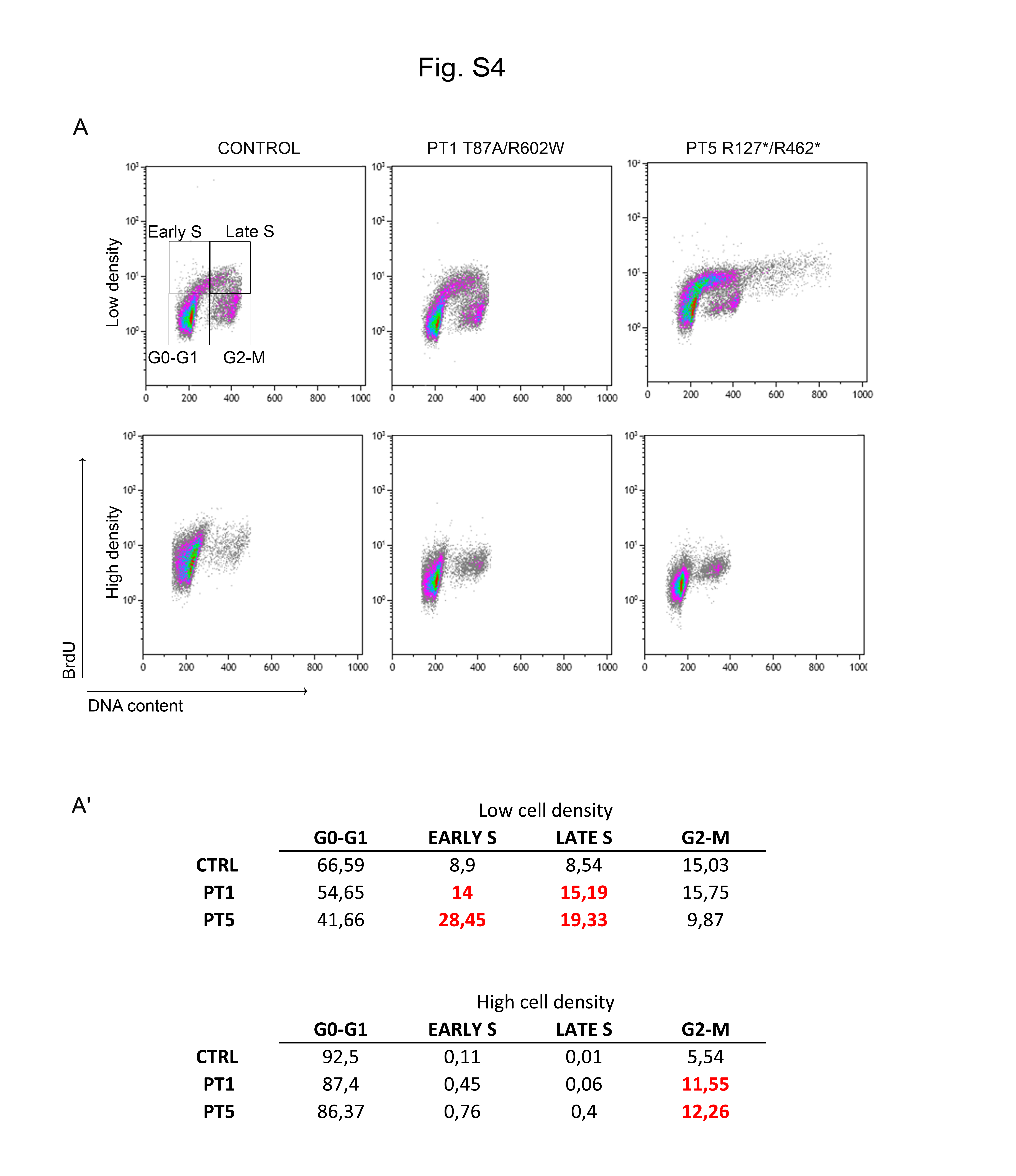

Supplement: S4 Fig — (A) Cell cycle analysis by flow cytometry of control and patient fibroblasts cultivated in low (top) and high cell density followed for 48 hours of serum starvation (bottom). Cells in S-phase stage were labeled with BrdU and DNA content was determined by propidium iodide staining. (A’) Table presenting the average percentage of cells in each phase of cell cycle, in low (top) and high (bottom) cell density conditions. (TIF) [file pgen.1005894.s004.tif]

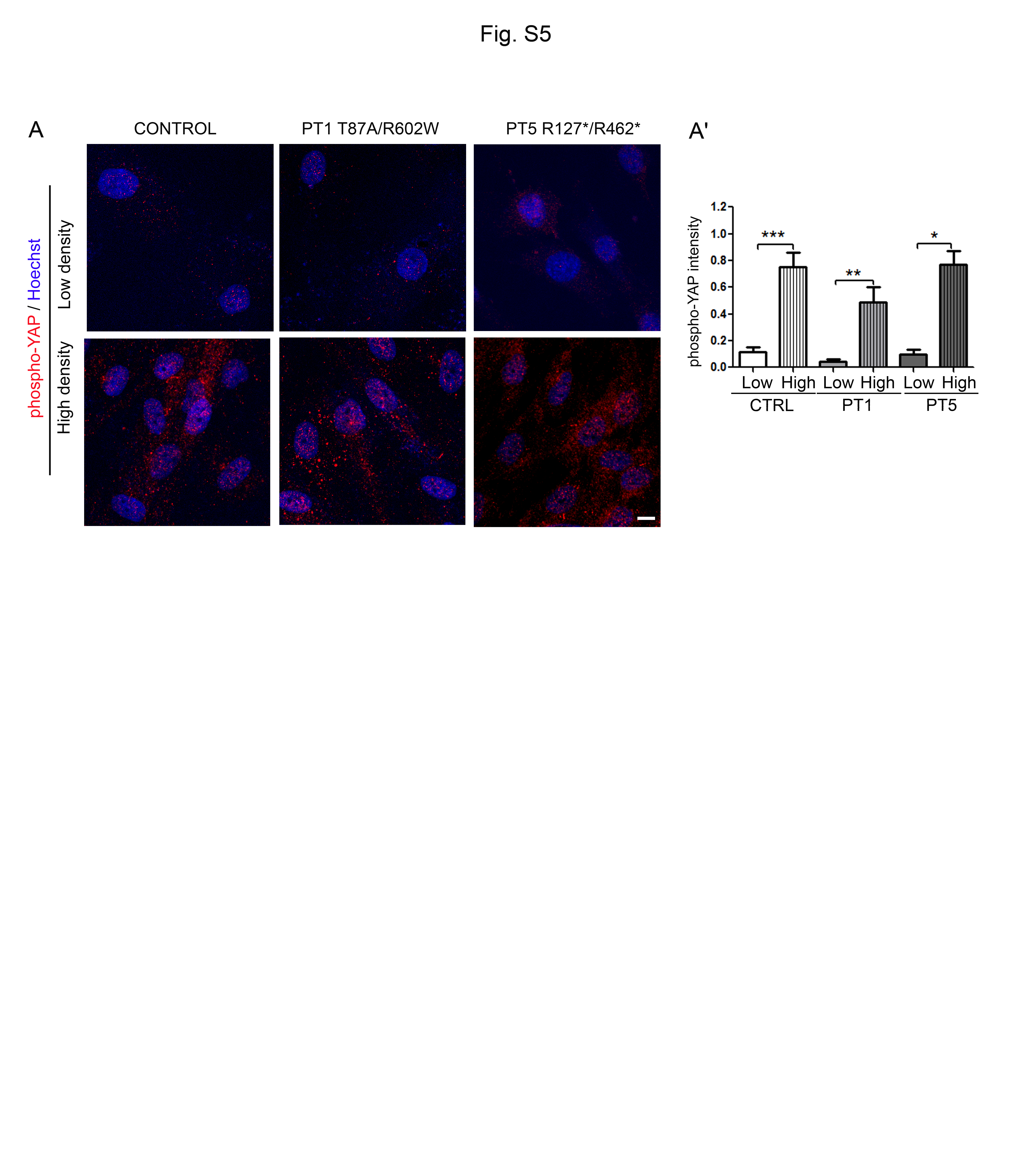

Supplement: S5 Fig — (A) Control and patient fibroblasts were fixed after 2 days (low cell density) or 6 days of culture in standard medium followed by 2 days of serum starvation (high cell density). Cells were stained with anti phospho-YAP antibody (red) and nuclei (Hoechst, blue). Scale bar, 10 μm. (A’) Quantification of phospho-YAP staining. *p < 0.05, **p < 0.01, calculated by Kruskall-Wallis test. (TIF) [file pgen.1005894.s005.tif]

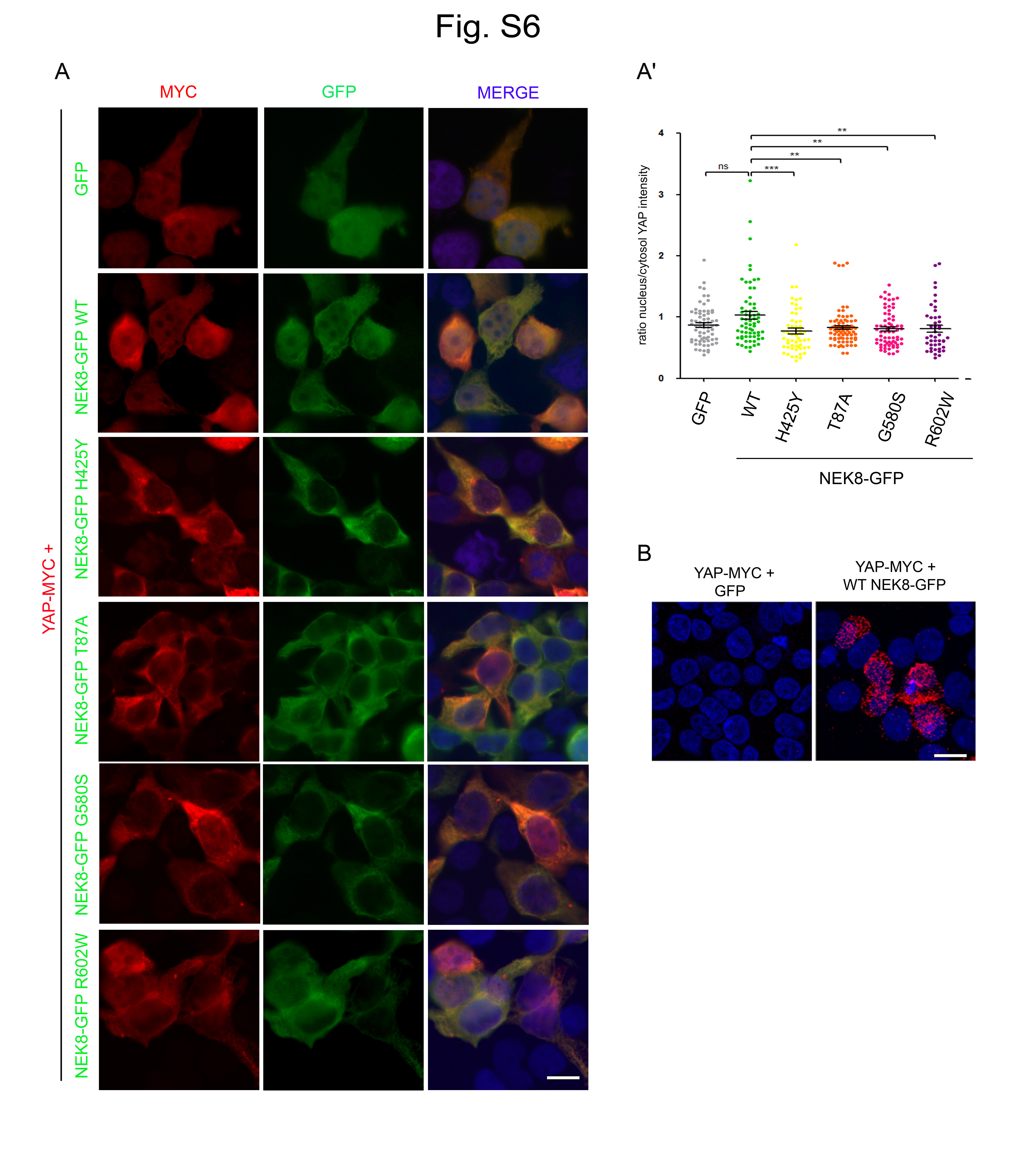

Supplement: S6 Fig — (A) HEK293T cells were co-transfected with WT or mutated NEK8-GFP and YAP-MYC constructs, fixed after 48 hours and stained for GFP (green) and MYC (red). Scale bar, 10 μm. (A’) Graph representing the ratio between nuclear and cytosolic YAP intensities, based on three independent experiments. ** p < 0.01, *** p < 0,001, calculated via Bonferroni post-hoc tests following ANOVA. (B) 48h after transfection, cells were fixed and a proximity ligation assay was performed using the appropriate anti-GFP and anti-myc antibodies, showing that YAP and NEK8 WT are in close vicinity. Scale bar, 10 μm. (TIF) [file pgen.1005894.s006.tif]

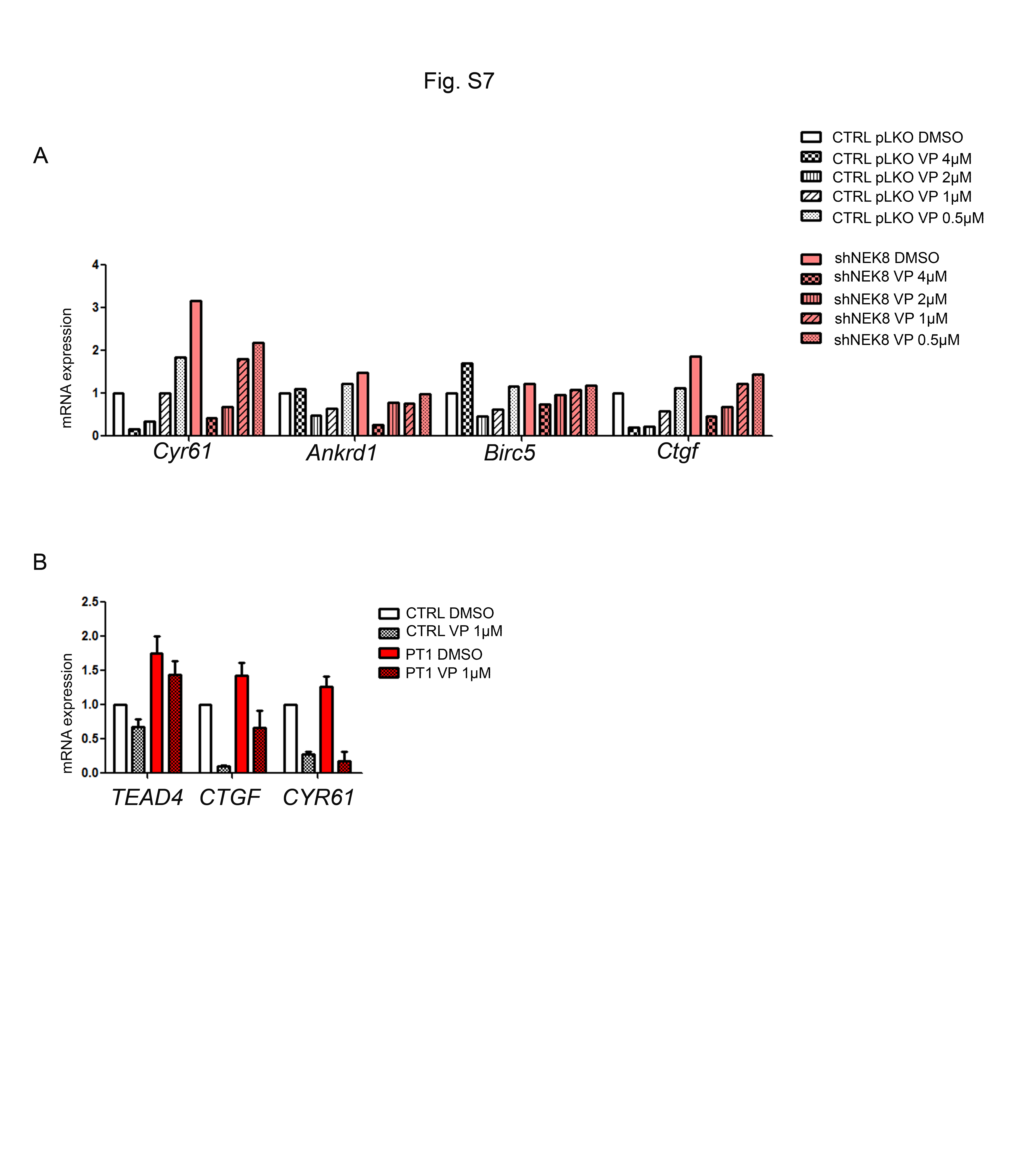

Supplement: S7 Fig — qPCR analyses of YAP target gene expression in DMSO- and Verteporfin (VP)-treated control (pLKO) and shNEK8 mIMCD3 cells (A), as well as in control and patient (PT1) fibroblasts (B). In both cell lines, NEK8 mutations lead to upregulation of YAP target genes, which is blocked upon Verteporfin treatment. (TIF) [file pgen.1005894.s007.tif]

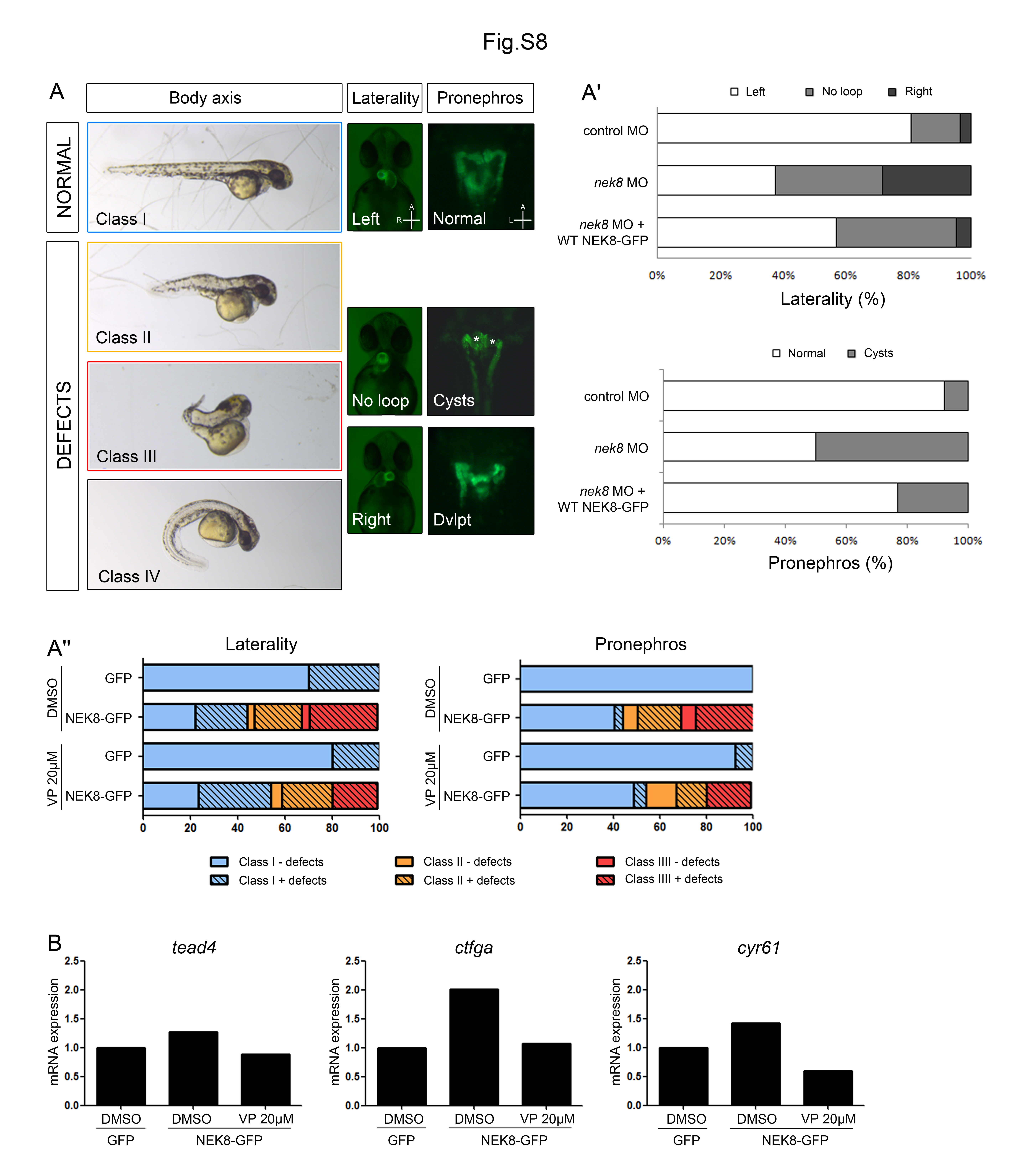

Supplement: S8 Fig — (A) Representative images of body axis, laterality (heart looping) and pronephros defects observed in zebrafish embryos. Four classes have been determined depending of the body shape, class I (blue) for normal embryos, class II (orange) for embryos with shortened axis, class III (red) for embryos with severely shortened and dorsally curved body axis, and class IV (black, only observed with nek8 MO) with ventrally curved body axis. Laterality defects encompass no looped and right-sided hearts compare to normal left-sided heart. Ventral views, anterior to the top. Pronephros defects encompass cystic glomeruli (asterisks) and developmental (Dvlpt) abnormalities. Dorsal views, anterior to the top. Tg(cmlc2:GFP) and Tg(wt1b:GFP) transgenic lines were used to observe heart looping and pronephros morphology, respectively. (A’) Graphs representing the proportions of embryos presenting laterality defects (top panel) and pronephric cysts (bottom panel) in control MO-, nek8 MO- and nek8 MO/human NEK8-GFP RNA-injected embryos. (A”) Graphs representing the proportions of embryos presenting laterality (left panel) and pronephros (right panel) defects (dashed bars) within each class of body axis shape, in control GFP and human NEK8-GFP RNA-injected embryos, treated with DMSO or Verteporfin (VP, 20 μM) from 90% epiboly to 34 hpf. (B) qPCR analysis of Yap target gene expression, tead4, ctgfa, cyr61, in human NEK8-GFP RNA-injected embryos treated with DMSO or Verteporfin (VP, 20 μM) compare to control GFP RNA injected embryos. (TIF) [file pgen.1005894.s008.tif]

## Slide 1
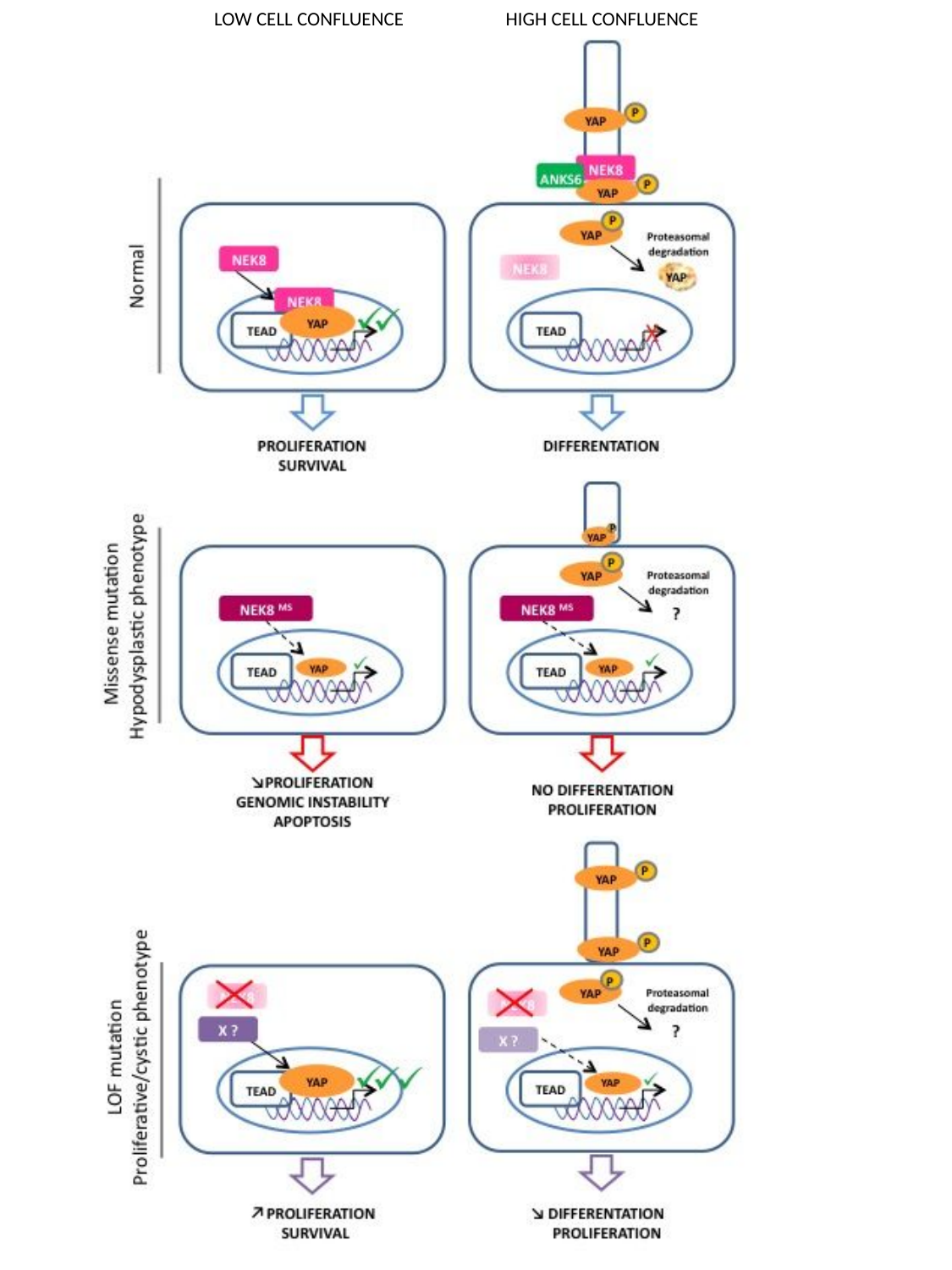

LOW CELL CONFLUENCE
HIGH CELL CONFLUENCE

Supplement: S9 Fig — In control cells at low cell density, NEK8 participates by its nuclear localization in insuring an efficient YAP-dependent transcriptional activity, allowing proliferation and cell survival. At high cell density, the Hippo pathway is activated and the total amount of NEK8 protein decreases through proteasomal degradation. We suggest that a decrease of NEK8 protein level facilitates the degradation of cytosolic YAP, stopping transcription of target genes. In parallel, NEK8 is targeted at the INVS compartment into the primary cilium, promoting the recruitment of both phospho-YAP and ANKS6 at the cilia. Altogether, cellular signals converge in the inhibition of proliferation in favour of differentiation. In patient cells at low cell density, NEK8 missense mutations prevent the nuclear localization of NEK8, thus causing a reduction of nuclear YAP localization and activity. As a consequence, patient cells fail to proliferate as much as control cells while undergoing both apoptosis and genomic instability. We hypothesize that NEK8 missense mutations also affect NEK8 proteasomal degradation, which participates in the maintenance of YAP into the nucleus and a sustained low level of transcriptional activity/proliferation when cells reach confluence. In parallel, missense mutations induce ciliogenesis defects and prevent NEK8 to localize at the cilium, resulting in defective INVS compartment integrity and loss of phospho-YAP at the cilium. Consequently, induction of differentiation is severely altered. In presence of NEK8 loss-of-function mutations, YAP nuclear translocation occurs under the control of (an)other, unidentified factor(s), resulting in a high level of proliferation. At high cell density, absence of NEK8, mimicking degradation of the protein that normally occurs, allows a reduction of the pool of nuclear YAP. However, as in the context of missense mutations, some YAP remains in the nucleus and proliferation is not arrested. Absence of NEK8 does not prevent cilium [file pgen.1005894.s009.pptx]

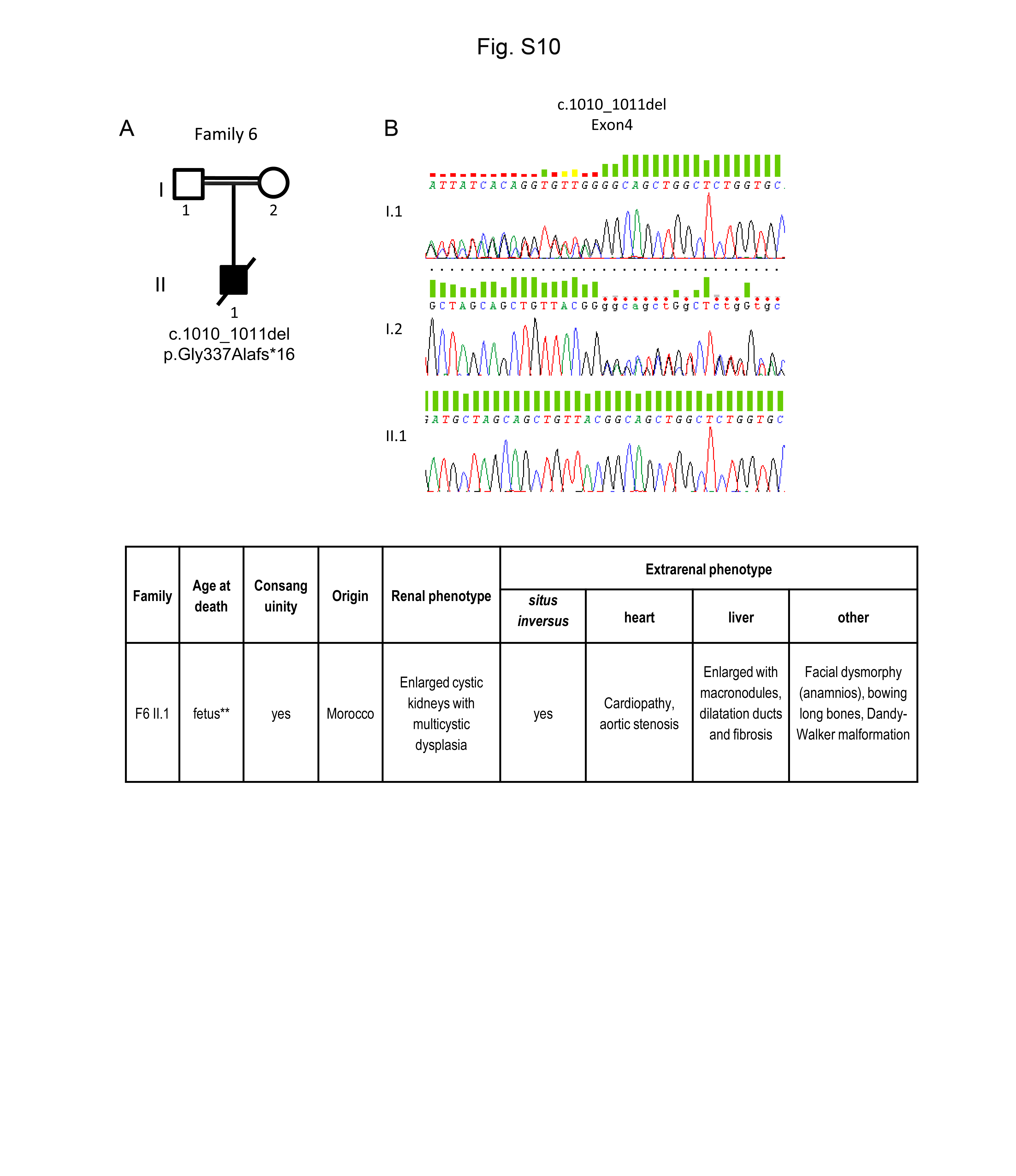

Supplement: S10 Fig — (A-B) Pedigree (A) and chromatograms (B) of family 6 identified with ANKS6/NPHP16 mutation. The mutation is numbered according to the human cDNA (NM_173551). Position +1 corresponds to the A of ATG. Abbreviations in the table are: CS, consanguinity; **Termination of pregnancy at 28 weeks of gestation. (TIF) [file pgen.1005894.s010.tif]
